# Traceless Solid-Phase Synthesis of Ketones via Acid-Labile Enol Ethers: Application in the Synthesis of Natural Products and Derivatives

**DOI:** 10.3390/molecules24071406

**Published:** 2019-04-10

**Authors:** Eva Schütznerová, Anna Krchňáková, Viktor Krchňák

**Affiliations:** 1 Department of Organic Chemistry, Faculty of Science, Palacký University, 17. listopadu 12, 771 46 Olomouc, Czech Republic; eva.schutznerova@upol.cz; 2 Department of Chemistry and Biochemistry, 251 Nieuwland Science Center, University of Notre Dame, Notre Dame, IN 46556, USA; viktor@torviq.com

**Keywords:** solid-phase synthesis, ketone, traceless synthesis, natural products, enol ethers

## Abstract

In solid-phase organic synthesis, Wang resin is traditionally used for the immobilization of acids, alcohols, phenols, and amines. We report the use of Wang resin for the traceless synthesis of ketones via acid-labile enol ethers. We demonstrate the practicality of this synthetic strategy on the solid-phase synthesis of pyrrolidine-2,4-diones, which represent the core structure of several natural products, including tetramic acid. Base-triggered condensation of pyrrolidine-2,4-diones yielded 4-hydroxy-1,1′,2′,5-tetrahydro-2*H*,5′*H*-[3,3′-bipyrrole]-2,5′-diones.

## 1. Introduction

Solid-phase synthesis is a very attractive methodology for the time-efficient synthesis of diverse organic molecules [1,2,3,4]. The initial step in the entire synthetic sequence is the selection of the appropriate linker for the immobilization of the first building block. The Wang linker [5] is the most commonly used acid-labile linker, and it has been used to immobilize carboxylic acids, alcohols, phenols, and amines [6,7]. Typically, after finishing the synthesis, the product is released from the resin and the functional group that was initially used for immobilization will remain attached to the product. This functional group, referred to as the trace of the linker, may be an inherent part of the target molecules (peptides are the best examples), but for the synthesis of organic molecules that do not share a common functional group, the trace of the linker is undesirable. Therefore, numerous synthetic routes have been devised that enabled the synthesis to be performed in a traceless manner; heterocycles are undoubtedly the highly representative examples [8]. Here, we expand the application of Wang resin to the novel, traceless synthesis of ketones from acid-labile enol ethers, which were prepared via the Wittig reaction of resin-bound esters.

Enol ethers represent valuable synthons in organic synthesis, and numerous methods for the synthesis of enol ethers have been reported; however, the Wittig olefination of esters is used rarely [9]. This ’nonclassical’ Wittig reaction of carboxylate esters suffers from sluggish reactivity due to the low electrophilicity of the carbonyl carbon towards phosphoranes compared to the electrophilicity of aldehydes and ketones [10]. The reactions typically require the use of microwave irradiation (conventional heating reportedly did not yield any product) [11] or reactive phosphoranes such as cyanomethylenetrimethylphosphorane [12]. An alternative approach to enol ethers is the alkylidenation of ester carbonyls with metal carbene complexes [13] used, for example, in traceless solid-phase synthesis of indoles [14,15].

On the other hand, the intramolecular Wittig cyclization of phosphonium salt proceeded smoothly, and this reaction was successfully used by Hercouet and Le Corre in 1979 for the synthesis of dihydrofurans and dihydropyrans [16,17], and later, this technique was used for the preparation of carbocycles [18,19,20] and heterocycles such as 2-alkylthiobenzimidazoles [21], indoles [22], 2-quinolones [23], and, recently, 4-alkoxy-1,5-dihydro-2*H*-pyrrol-2-ones [24,25].

Not surprisingly, although only a limited number of reports have described the application of enol ethers in the synthesis of ketones, the preparation of ketones from vinyl ethers using a Grignard reagent was reported in 1955 [26]. The hydrolysis of vinyl ethers has been studied on numerous occasions [27,28,29]; however, this technique has not been applied for general preparative use. Among the few recent reports in this area, silyl enol ethers were enantioselectively converted to ketones by Cheon and coworkers [30,31]. Ketones were prepared by the palladium-catalyzed regioselective arylation of vinyl ethers [32] and by the hydrolysis (MeOH/aq HCl, reflux) of vinyl ethers [33].

To summarize the prior work, neither the synthesis of enol ethers via a Wittig olefination nor the use of enol ethers in the synthesis of ketones is a method of choice for ketone synthesis. Here, we report a simple and practical synthesis of acid-labile Wang resin-bound enol ethers via the Wittig olefination of carboxylate esters and subsequent acid-mediated traceless release of the ketones from the resin.

## 2. Results and Discussion

To demonstrate the practical use of a Wang linker for the traceless synthesis of pharmacologically relevant ketones, we report the synthesis of pyrrolidine-2,4-diones. Pyrrolidine-2,4-dione, the core structure of tetramic acid, was selected as a pharmacologically relevant structure found in natural products [34,35]. Numerous chemical routes for the preparation of tetramic acid and its derivatives have been developed, and the reported syntheses have been reviewed [35,36,37].

### 2.1. Synthesis

**Scheme 1:**
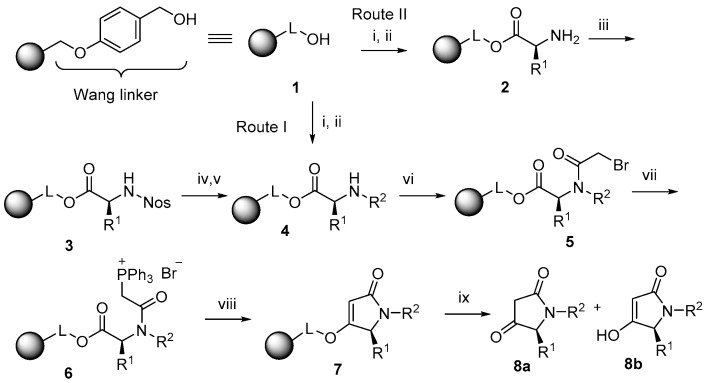
Traceless solid-phase synthesis of pyrrolidine-2,4-dione. Reagents and conditions: (i) Fmoc-amino acid-OH (Fmoc–AA–OH), *N,N′-*diisopropylcarbodiimide (DIC), hydroxybenzotriazole (HOBt), 4-(dimethylamino)pyridine (DMAP), dichloromethane (DCM)/ dimethylformamid (DMF) (1:1), room temperature (rt), overnight; (ii) piperidine/DMF (1:1), rt, 15 min; (iii) Ns-Cl, 2,6-lutidine, DCM, rt, 2 h; (iv) alcohol, PPh_3_, diisopropyl azodicarboxylate (DIAD), anhydrous THF, rt, 2 h; (v) mercaptoethanol, DBU, DMF, rt, 5 min; (vi) bromoacetic acid, DIC, DCM, 5 min, then the precipitated diisopropyl urea (DIU) was removed by filtration, *N,N*-diisopropylethylamine (DIEA) was added, and the solution was transferred to the reaction vessel with the resin, rt, 1 h; (vii) PPh_3_, anhydrous NMP, rt, overnight; (viii) TEA, anhydrous NMP, rt, 2–48 h, or DBU, anhydrous NMP, 60 °C, overnight (see the Supplementary Materials Table S1 for details); (ix) TFA/DCM 1:1, rt, 1 h. Abbreviations:; AA: amino acid; DIC: *N,N′*-diisopropylcarbodiimide; HOBt = hydroxybenzotriazole; DMAP: 4-(dimethylamino)pyridine; DCM: dichloromethane; DMF: dimethylformamide; rt: room temperature; DIAD: diisopropyl azodicarboxylate; DIU: diisopropyl urea; DIEA: *N,N*-diisopropylethylamine.

**Figure 1:**
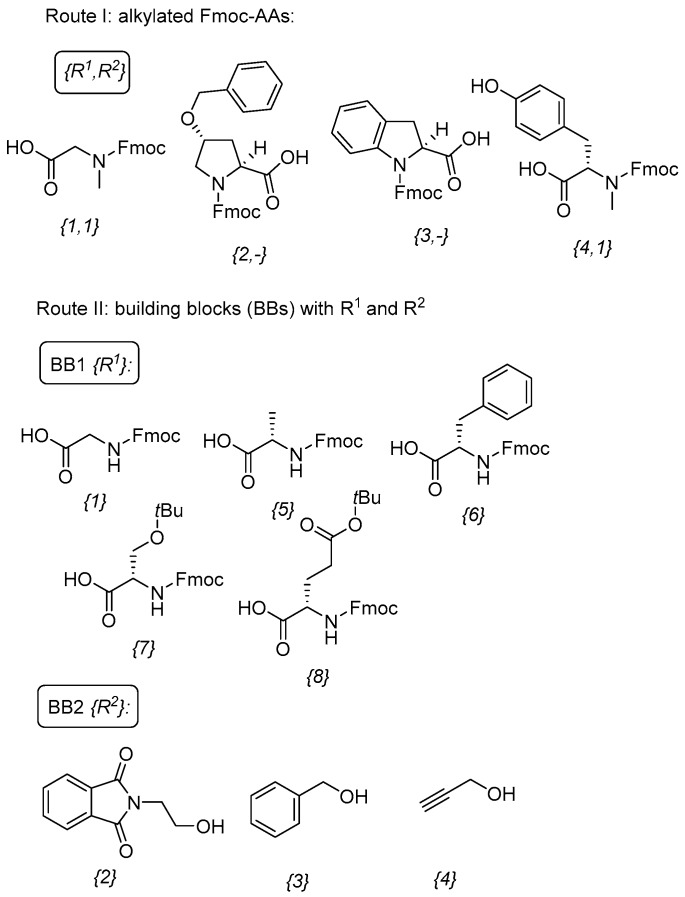
List of building blocks (BBs) used in synthesis.

The assembly of the acyclic precursor was efficiently carried out on a solid phase using optimized protocols for the individual transformations. Wang resin **1** was acylated with the *N*-[(9*H*-Fluoren-9-ylmethoxy)carbonyl] (Fmoc) *N*-alkyl amino acids (sarcosine, 4-OBzl-proline (Hyp(Bzl)), 2-indolinecarboxylic acid (Idc), and methyltyrosine (O*t*Bu)), and the Fmoc protecting group was cleaved to yield resin **4** (Scheme 1, route I). Because of the limited number of commercially available *N*-alkyl amino acids, we also evaluated an alternative route using resin *N*-alkylation. Thus, Wang resin **1** was esterified with Fmoc–amino acids, the Fmoc group was cleaved, and amine **2** was reacted with 4-nitrobenzenesulfonyl chloride (Ns-Cl) (resin **3**) to facilitate Mitsunobu alkylation with alcohols [38], which introduced the *N*-substituent (Scheme 1, route II). This reaction sequence was designed to enhance the diversity of compounds available with this method. Removal of the Ns group yielded secondary amine **4**. Resin-bound amine **4** was then acylated with bromoacetic acid (resin **5**) and reacted with PPh_3_ to form phosphonium salt **6**. The resin-bound phosphonium salt was not isolated, and the LC/MS analysis of trifluoroacetic acid (TFA)-cleaved sample revealed the expected molecular ion in all prepared compounds. The addition of trimethylamine (TEA) or 1,8-diazabicyclo[5.4.0]undec-7-ene (DBU) in *N*-methyl-2-pyrrolidone (NMP) triggered the Wittig olefination. The olefination of the ester proceeded smoothly at ambient temperature. TFA exposure released products **8**. Crude products were isolated and then purified by reversed-phase (RP) HPLC in an acidic mobile phase (aqueous formic acid or TFA), and tetramic acids **8** were fully characterized (Table 1). The structures of the building blocks are listed in Figure 1.

To address the presence of tautomers **8a** and **8b**, we collected their NMR spectra in CDCl_3_ and DMSO-*d_6_*. As expected and in agreement with previously reported data [39,40], we observed the presence of one isomer (**8a**) in CDCl_3_ based on the diagnostic proton resonances corresponding to the two methylene protons of isomer **8a***{1,2}* (δ = 4.06 and 2.92 ppm) and the ketone carbon of isomer **8a***{1,2}* (δ = 203.0 ppm). However, the NMR spectrum acquired in DMSO-*d_6_* revealed the presence of a mixture of tautomers **8a** and **8b** (in an approximately 1:1 ratio) based on the diagnostic proton resonances corresponding to the methylene protons of isomer **8a***{1,2}* (δ = 3.89 and 2.91 ppm) and the olefinic proton of isomer **8b***{1,2}* (δ = 4.62 ppm). Moreover, the ^13^C NMR spectra unambiguously showed the presence of the ketone carbon of isomer **8a***{1,2}* (δ = 205.0 ppm) as well as the olefin carbon of isomer **8b***{1,2}* (δ = 93.4 ppm). The NMR spectra of all other compounds are presented in the Supplementary Materials.

### 2.2. Self-Condensation

**Scheme 2:**
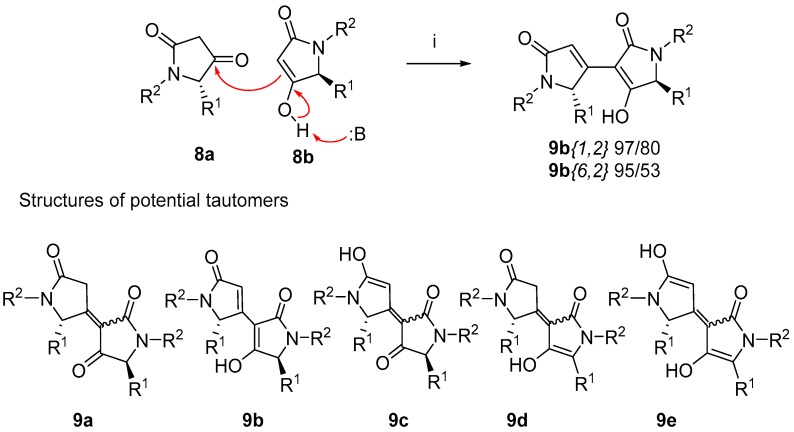
Self-condensed pyrrolidine-2,4-diones and possible tautomers. Reagents and conditions: (i) aqueous ammonium acetate buffer, MeCN, rt, 24 h.

Today’s search for new drugs is focused on the design and synthesis of compounds structurally resembling natural products, referred to as biology-oriented synthesis [41,42,43,44,45]. Because our synthetic route provides traceless access to ketones, we investigated their potential for self-condensation that would convert pyrrolidine-2,4-diones **8** to the natural product derivatives 4-hydroxy-1,1′,2′,5-tetrahydro-2*H*,5′*H*-[3,3′-bipyrrole]-2,5′-diones **9** (Scheme 2). This condensed product has not been exploited as a potential pharmacologically relevant structure, although an analogous self-condensation was reported as a side-reaction in 1985 [39]. It is important to emphasize that the 2-substituted pyrrolidine-2,4-diones do not form condensed products.

In a search for reaction conditions for condensation, we found that pyrrolidine-2,4-diones **8** undergo self-condensation in basic solution at ambient temperature and form bisheterocycles **9**. Exposure of purified pyrrolidine-2,4-dione **8***{1,2}* to 10 mM aqueous ammonium acetate buffer in acetonitrile triggered quantitative conversion within 24 h, and compound **9***{1,2}* was isolated in 80% yield. We also purified crude compound **8***{1,2}* by reversed-phase HPLC in an acetonitrile/aqueous ammonium acetate buffer and isolated clean **9***{1,2}* in an overall 53% yield (without purification of the **8***{1,2}*). The ^1^H and ^13^C NMR spectra and HRMS analysis confirmed the bisheterocyclic structure of **9***{1,2}*, formed by the self-condensation of two molecules of tetramic acid.

Based on the results described above, we also tested the stability of purified pyrrolidine-2,4-dione **8***{1,2}*. The compound was stable and no spontaneous conversion into **9***{1,2}* was observed after storage of the HPLC-purified sample in DMSO at 4 °C (in a refrigerator) for one month.

### 2.3. Structure Determination

Base-triggered condensed product **9** can exist as several tautomers (Scheme 2). To determine the structure of the tautomer present in solution, NMR spectra were measured both in DMSO-d_6_ and CDCl_3_, and they exhibited analogous patterns. The proton NMR spectrum of **9***{1,2}* showed two methylene singlets (δ = 4.43 and 3.99) and one singlet corresponding to an olefinic proton (δ = 6.15). These resonances indicated the presence of tautomer **9b** or **9c**. The ^13^C NMR spectra did not show the presence of a ketone carbon (δ = 205.0 in the case of compound **8**), eliminating tautomer **9c**. Analogous findings were observed with compound **9***{5,3}*, which exhibited one diagnostic olefin singlet and two quartets coupled to two methyl groups, suggesting the same type of tautomer **9b**. The LC/MS analysis and NMR spectra of **9** also did not indicate the presence of any diastereomer, confirming that the optical integrity of the amino acid chiral carbon was preserved.

## 3. Conclusion

We demonstrated a general and novel application of a Wang linker for the traceless solid-phase synthesis of ketones from acid-labile enol ethers. The synthesis of tetramic acid derivatives, including self-condensed 4-hydroxy-1,1′,2′,5-tetrahydro-2*H*,5′*H*-[3,3′-bipyrrole]-2,5′-diones, highlighted the practical use of this protocol for the synthesis of pharmacologically relevant natural products. The advantages of solid-phase synthesis enabled time-efficient synthesis and preparation of products with any combination of building blocks. An extension of this synthetic route to the synthesis of other natural products and its application in self-condensation is in progress and will be reported in due course.

## 4. Experimental Procedures

### 4.1. General Information

Solvents were used without further purification. The Wang linker (100–200 mesh, 1% DVB, 0.9 mmol/g) was used. Synthesis was carried out on Domino Blocks [46] in disposable polypropylene reaction vessels. The volume of wash solvent was 10 mL per 1 g of resin. For washing, resin slurry was shaken with the fresh solvent for at least 1 min before changing the solvent. After adding a reagent solution, the resin slurry was manually vigorously shaken to break any potential resin clumps. Resin-bound intermediates were dried by a stream of nitrogen for prolonged storage and/or quantitative analysis.

### 4.2. Esterification with Fmoc–AA (Resins **2** and **4**)

Resin **1** (1 g) was washed with DCM (3 × 10 mL) and treated with a solution of Fmoc–amino acid (2 mmol) and HOBt⸱H_2_O (306 mg, 2 mmol), DMAP (61 mg, 0.5 mmol), and DIC (312 μL, 2 mmol) in 10 mL of DMF/DCM (1:1), and the reaction slurry was shaken overnight at room temperature. The resin was washed with DMF (3 × 10 mL) and DCM (5 × 10 mL).

Quantification of Resin Loading: A sample of resin was washed 5 times with DCM, 3 times with MeOH, and then dried with nitrogen. A 10-mg portion of resin was cleaved with 50% TFA in DCM for 30 min. The cleavage cocktail was evaporated by a stream of nitrogen, and the cleaved compound was extracted into 1 mL of MeOH. This sample of Fmoc derivate was analyzed by LC/MS, and the quantity was determined by comparison with a standard (Fmoc–Ala–OH; concentration: 1 mg/mL). The loading of the resin was determined by external standard method by integration of the UV response at 300 nm.

Fmoc deprotection: The resin (1 g) was washed with DCM (3 × 10 mL) and DMF (3 × 10 mL) and treated with a solution of 50% piperidine in DMF (10 mL) for 15 min at room temperature. The resin was thoroughly washed with DMF (5 × 10 mL) and DCM (3 × 10 mL).

### 4.3. Reaction with Ns-Cl (Resin **3**)

Resin **2** (1 g) was swollen with DCM, washed with DCM (3 times), and a solution of Ns-Cl (3 mmol, 663 mg) and 2,6-lutidine (1 mmol, 382 µL) in DCM (10 mL) was added. The reaction slurry was shaken at rt for 2 h, and then the resin was washed with DCM (3 times). 

### 4.4. Fukuyama N-Alkylation (Resin **4**)

The Ns resin **3** (1 g) was swollen in anhydrous THF, a solution of alcohol (2 mmol) with PPh_3_ (2 mmol, 524 mg) in anhydrous THF (8 mL) was added to the resin, and the slurry was left in the freezer for 30 min. Subsequently, cooled DIAD (2 mmol, 393 µL) in anhydrous THF (2 mL) was added to the resin, and the reaction mixture was shaken at rt for 2 h. The resin was then washed with THF (3 times) and DCM (3 times).

Resin was swollen in DCM and washed with DMF (3 times), and the Ns group was cleaved with 2-mercaptoethanol (6 mmol, 420 µL) and DBU (2 mmol, 300 µL) in DMF (10 mL) for 5 min. The resin was washed with DMF (3 times) and DCM (3 times).

### 4.5. Acylation with Bromoacetic Acid (Resin **5**)

A solution of bromoacetic acid (700 mg, 5 mmol) was prepared in another syringe with a frit, and DIC (386 μL, 2.5 mmol) was added. After 5 min, DIU was removed by filtration, lutidine (292 μL, 2.5 mmol) was added, and the solution was transferred to the syringe with resin **4**. The slurry was shaken for 1 h at room temperature. The resin was washed with DCM (3 × 10 mL).

### 4.6. Preparation of the Triphenylphosphonium Salt (Resin **6**)

Resin **5** (1 g) was washed with DCM (3 × 10 mL) and anhydrous NMP (3 × 10 mL). A solution of triphenylphosphine (1.05 g, 4 mmol) in anhydrous NMP (10 mL) was added to the resin, and the slurry was shaken overnight at room temperature. The resin was washed with NMP (3 × 10 mL) and DCM (3 × 10 mL).

### 4.7. Wittig Olefination (Resins **7**)

Resin-bound triphenylphosphonium salt **6** (500 mg), prepared according to a recently published procedure [24,25] was washed with DCM (3 × 10 mL) and anhydrous NMP (3 × 10 mL). A solution of TEA (70 μL, 0.5 mmol) or DBU (71 μL, 0.5 mmol) in anhydrous NMP (5 mL) was added to the resin, and the slurry was shaken at room temperature (see Table S1 in the Supplementary Materials). The resin was washed with NMP (3 × 10 mL) and DCM (3 × 10 mL).

### 4.8. Cleavage from the Resin (Compounds **8**)

The cyclized resin **7** (250 mg) was washed with DCM (3 × 10 mL). The resin was treated with 3 mL of a solution of 50% TFA in DCM for 1 h at room temperature. The TFA solution was collected, and then the resin was washed with 10% TFA in DCM (5 mL) and DCM (5 mL), and the combined extracts were concentrated under a stream of nitrogen. The crude product was dissolved in 3 mL of MeOH and purified by semipreparative RP HPLC in MeCN/aqueous TFA or formic acid.

### 4.9. Self-Condensation (Compounds **9**)

Two compounds, **8***{1,2}* and **8***{6,2}*, purified by RP HPLC in MeCN/aqueous 0.1% TFA or formic acid, were dissolved in 600 μL of DMSO and 5 mL of 10 mM aqueous ammonium acetate was added. The solution was left at rt overnight, and then condensed compounds **9** were purified in MeCN/10 mM aqueous ammonium acetate (Table S2). The remaining compounds were subjected to self-condensation without purification; however, the self-condensed products **9** were purified.

## Figures and Tables

**Table 1:** List of prepared compounds **8** and **9** (purity/yield).

Entry	Compound	Route	R^1^	R^2^	Purity of 8 (%)	Yield of 8 (%) ^b^	Yield of 9 (%) ^c^
1	*{1,1}*	I	H	Me	85	NI ^d^	47
2	*{1,2}*	II	H	PhthN(CH_2_)_2_	81	70	53
3	*{2,-}*	I	Hyp(Bzl) ^a^		81	NI ^d^	37
4	*{3,-}*	I	Idc ^a^		52	31	NT ^e^
5	*{4,1}*	I	CH_2_C_6_H_4_OH	Me	77	25	NT ^e^
6	*{5,3}*	II	Me	Bn	56	NI ^d^	23
7	*{6,2}*	II	Bn	PhthN(CH_2_)_2_	72	30	49
8	*{6,4}*	II	Bn	CH_2_CCH	88	21	NT ^e^
9	*{7,2}*	II	CH_2_OH	PhthN(CH_2_)_2_	68	52	NT ^e^
10	*{8,2}*	II	(CH_2_)_2_CO_2_H	PhthN(CH_2_)_2_	52	21	NT ^e^

Note: In the compound labeling, the first digit refers to the R^1^ substituent and the second digit refers to the R^2^ substituent; ^a^ compounds were prepared using cyclic amino acids Hyp(Bzl) for *{2,-}* and Idc for *{3,-}*; ^b^ HPLC purification in acetonitrile/aqueous TFA or formic acid; ^c^ purification in acetonitrile/aqueous ammonium acetate; ^d^ NI, not isolated, crude compounds **8** were converted to **9**; ^e^ NT, not tested.

## Supplementary Materials

The following are available online: ^1^H and ^13^C NMR spectral data and figures of all compounds.
