# Supplementary material for: Traceless Solid-Phase Synthesis of Ketones via Acid-Labile Enol Ethers: Application in the Synthesis of Natural Products and Derivatives
Source: Molecules. 2019 Apr 10;24(7):1406. doi: 10.3390/molecules24071406 (PMC6479686; doi:10.3390/molecules24071406)

Supplementary Materials

Article

Traceless Solid-Phase Synthesis of Ketones via Acid-Labile Enol Ethers: Application in the Synthesis of Natural Products and Derivatives

Eva Schütznerová [1], Anna Krchňáková [2] and Viktor Krchňák [1,2,*]

[1] Department of Organic Chemistry, Faculty of Science, Palacký University, 17. listopadu 12, 771 46 Olomouc, Czech Republic; eva.schutznerova@upol.cz

[2] Department of Chemistry and Biochemistry, 251 Nieuwland Science Center, University of Notre Dame, Notre Dame, Indiana 46556, United States; viktor@torviq.com

* Correspondence: vkrchnak@nd.edu

Table of Contents

1. Table S1………………………………………………………………………………… S2

2. Table S2………………………………………………………………………………… S2

3. General Information……………………………………………………………….. S3

4. Analytical data……………………………………………………………………….. S5

5. ^{1}H and ^{13}C NMR Spectra …………………………………………………..…… S11

Table S1: Base-catalyzed Wittig olefination to compounds **7**.

Entry	Cmpd	Route	R^1	R^2	Base	Conditions
1	*{1,1}*	I	H	Me	0.1 M DBU	60 °C, 16 h
2	*{1,2}*	II	H	$PhthN(CH_2)_2$	0.1 M TEA	rt, 48 h
3	*{2,-}*	I	Hyp(Bzl)[a]		0.1 M DBU	60 °C, 48 h
4	*{3,-}*	I	Idc[a]		0.1 M TEA	rt, 48 h
5	*{4,1}*	I	$CH_2C_6H_4OH$	Me	0.1 M TEA	rt, 48 h
6	*{5,3}*	II	Me	Bn	0.1 M TEA	rt, 2 h
7	*{6,2}*	II	Bn	$PhthN(CH_2)_2$	0.1 M TEA	rt, 48 h
8	*{6,4}*	II	Bn	CH_2CCH	0.5 M TEA	rt, 16 h
9	*{7,2}*	II	CH_2OH	$PhthN(CH_2)_2$	0.1 M TEA	rt, 48 h
10	*{8,2}*	II	$(CH_2)_2CO_2H$	$PhthN(CH_2)_2$	0.1 M TEA	rt, 48 h

Note: [a]cyclic amino acids

Table S2: Self-condensation of purified compounds **8** to **9** in ammonium acetate buffer.

Entry	Cmpd	R^1	R^2	Purity **8** [%]	Yield of **8** [%][a]	Purity **9** [%][b]	Yield **9** [%]
1	*{1,2}*	H	$PhthN(CH_2)_2$	81	70	97	80
2	*{6,2}*	Bn	$PhthN(CH_2)_2$	72	30	95	53

Note: [a]Crude compounds **8** were purified by RP HPLC in MeCN/aqueous 0.1% TFA or formic acid; [b]Procedure for self-condensation: solution of compounds **8** (14.1 mg for *{1,2}*, 11.9 mg for *{6,2}*) in 600 μL of DMSO was added to 5 mL of 10 mM aqueous ammonium acetate and left at rt overnight, then compounds **9** were purified in MeCN/10 mM aqueous ammonium acetate.

General Information

Solvents were used without further purification. The Wang linker (100-200 mesh, 1% DVB, 0.9 mmol/g) was used. Synthesis was carried out on Domino Blocks (www.torviq.com) in disposable polypropylene reaction vessels.

The volume of wash solvent was 10 mL per 1 g of resin. For washing, resin slurry was shaken with the fresh solvent for at least 1 min before changing the solvent. After adding a reagent solution, the resin slurry was manually vigorously shaken to break any potential resin clumps. Resin-bound intermediates were dried by a stream of nitrogen for prolonged storage and/or quantitative analysis.

For the LC/MS analysis a sample of resin (~5 mg) was treated by 50% TFA in DCM, the cleavage cocktail was evaporated by a stream of nitrogen, and cleaved compounds extracted into 1 mL of MeOH.

The LC/MS analyses were carried out using two instruments. The first one comprised a 3 x 50 mm C18 reverse phase column, 5 um particles. Mobile phases: 10 mM ammonium acetate in HPLC grade water (A) and HPLC grade acetonitrile (B). A gradient was formed from 5% to 80% of B in 10 minutes, flow rate of 0.7 mL/min. The MS electrospray source operated at capillary voltage 3.5 kV and a desolvation temperature 300 ^{o}C. The second instrument comprised a 2.1 x 50 mm C18 reverse phase column, 2.6 um particles, at 30°C and flow rate of 800 µL/min. Mobile phases: 10 mM ammonium acetate in HPLC grade water (A) and HPLC grade acetonitrile (B). A gradient was formed from 10% to 80% of B in 2.5 minutes; kept for 1.5 minute, flow rate of 0.8 mL/min. The column was re-equilibrated with 10% solution B for 1 minute. The APCI source operated at discharge current of 5 µA, vaporizer temperature of 400 °C and capillary temperature of 200 °C.

Purification was carried out on C18 reverse phase column 19 x 100 mm, 5 µm particles, gradient was formed from 10 mM aqueous ammonium acetate (acidic mobile phase: 0.1% aqueous TFA or formic acid) and acetonitrile, flow rate 20 mL/min.

All ^{1}H and ^{13}C NMR experiments were performed at magnetic field strengths of 9.39 T (with operating frequencies 399.78 MHz for ^{1}H and 100.53 MHz for ^{13}C) at ambient temperature (20 °C). ^{1}H spectra and ^{13}C spectra were referenced relative to the signal of DMSO (^{1}H δ = 2.49 ppm, ^{13}C δ = 39.50 ppm).

HRMS analysis was performed using LC-MS on an Orbitrap Elite high-resolution mass spectrometer (Dionex Ultimate 3000, Thermo Exactive plus, MA, USA) operating at positive full scan mode (120,000 FWMH) in the range of 100–1000 *m*/*z*. The settings for electrospray ionization were as follows: oven temperature of 150 °C and source voltage of 3.6 kV. The

acquired data were internally calibrated with diisooctyl phthalate as a contaminant in CH_3OH (*m*/*z* 391.2843). Samples were diluted to a final concentration of 0.1 mg/mL in H_2O and CH_3OH (50:50, v/v). Before HPLC separation (column Phenomenex Gemini, 50 × 2.00 mm, 3 μm particles, C18), the samples were injected by direct infusion into the mass spectrometer using an autosampler. The mobile phase was isocratic CH_3CN/IPA/0.01 M ammonium acetate (40:5:55) and flow 0.3 mL/min.

Analytical Data of Synthetic Compounds

2-(2-(2,4-Dioxopyrrolidin-1-yl)ethyl)isoindoline-1,3-dione 8a*{1,2}* and 2-(2-(4-hydroxy-2-oxo-2,5-dihydro-1H-pyrrol-1-yl)ethyl)isoindoline-1,3-dione 8b*{1,2}*

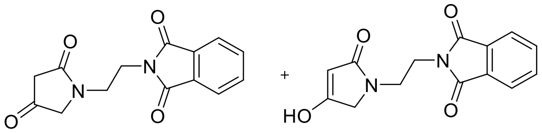

8a*{1,2}* **8b***{1,2}*

Yield 18.6 mg (70 %) of amorphous solid. Mixture of tautomers **8a***{1,2}* and **8b***{1,2}* in DMSO: ^{1}H NMR (400 MHz, DMSO-d_6) δ = 11.33 (s, 1 H), 7.92 - 7.77 (m, 8 H), 4.62 (s, 1 H), 4.00 (s, 2 H), 3.89 (s, 2 H), 3.79 (t, *J* = 5.5 Hz, 2 H), 3.69 (t, *J* = 5.3 Hz, 2 H), 3.65 - 3.57 (m, 2 H), 3.55 - 3.47 (m, 2 H), 2.91 (s, 2 H). ^{13}C NMR (101 MHz, DMSO-d_6) δ = 205.2, 172.4, 172.2, 169.7, 168.0, 167.7, 134.4, 134.3, 131.6, 131.6, 123.1, 123.0, 93.4, 56.8, 50.1, 41.3, 36.0, 34.6 Only tautomer **8a***{1,2}* in $CDCl_3$: ^{1}H NMR (400 MHz, $CDCl_3$) δ = 7.87 - 7.82 (m, 2 H), 7.75 - 7.71 (m, 2 H), 4.13 - 3.99 (m, 2 H), 3.95 - 3.90 (m, 2 H), 3.83 - 3.72 (m, 2 H), 2.92 (s, 2 H). ^{13}C NMR (101 MHz, $CDCl_3$) δ = 203.0, 169.7, 168.4, 134.2, 131.9, 123.5, 57.2, 41.3, 41.1, 34.8. HRMS (ESI-Orbitrap) *m/z* calcd for $C_{14}H_{12}N_2O_4$ [M+H]$^+$ 273.0870, found 273.0870.

(*S*)-9,9a-dihydro-1*H*-pyrrolo[1,2-*a*]indole-1,3(2*H*)-dione 8a*{3,-}* and (*S*)-1-hydroxy-9,9a-dihydro-3*H*-pyrrolo[1,2-*a*]indol-3-one 8b*{3,-}*

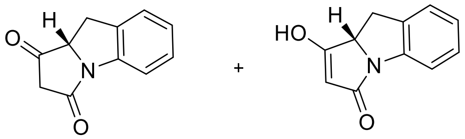

8a*{3,-}* **8b***{3,-}*

Yield 15.8 mg (31 %) of amorphous solid. Mixture of tautomers **8a***{3,-}* and **8b***{3,-}* in DMSO: ^{1}H NMR (400 MHz, DMSO-d_6) δ = 12.34 (br. s., 1 H), 7.43 (d, *J* = 7.8 Hz, 1 H), 7.31 - 7.13 (m, 5 H), 7.12 - 7.05 (m, 1 H), 7.00 (dt, *J* = 1.1, 7.4 Hz, 1 H), 5.11 - 5.05 (m, 1 H), 5.02 (t, *J* = 9.4 Hz, 1 H), 4.86 (s, 1 H), 3.79 - 3.69 (m, 1 H), 3.28 - 3.17 (m, 2 H), 3.15 - 2.97 (m, 3 H). ^{13}C NMR (101 MHz, DMSO-d_6) δ = 206.0, 179.5, 175.7, 169.7, 142.1, 140.6, 134.6, 133.3, 127.4, 125.4, 124.7, 123.6, 116.3, 116.0, 94.2, 70.0, 64.4, 46.1, 31.4, 29.9. HRMS (ESI-Orbitrap) *m/z* calcd for $C_{11}H_9NO_2$ [M+H]$^+$ 188.0706, found 188.0706.

(*S*)-5-(4-Hydroxybenzyl)-1-methylpyrrolidine-2,4-dione and 8a*{4,1}* and (*S*)-4-hydroxy-5-(4-hydroxybenzyl)-1-methyl-1,5-dihydro-2*H*-pyrrol-2-one 8b*{4,1}*

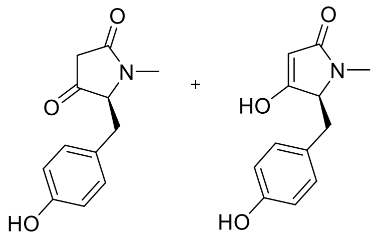

8a{4,1} **8b**{4,1}

Yield 21.6 mg (25 %) of amorphous solid. Mixture of tautomers **8a**{4,1} and **8b**{4,1} in DMSO: ^{1}H NMR (400 MHz, DMSO-d_6) δ = 11.39 (br. s., 1 H), 9.31 (s, 1 H), 9.18 (s, 1 H), 6.93 - 6.88 (m, 2 H), 6.88 - 6.83 (m, 2 H), 6.68 - 6.63 (m, 2 H), 6.62 - 6.58 (m, 1 H), 4.62 (s, 1 H), 4.15 (dt, *J* = 1.4, 4.6 Hz, 1 H), 4.06 (t, *J* = 4.4 Hz, 1 H), 2.85 (s, 3 H), 2.84 (d, *J* = 21.0 Hz, 1 H), 3.04 - 2.79 (m, 5 H), 2.71 (s, 3 H), 2.32 (d, *J* = 22.0 Hz, 1 H). ^{13}C NMR (101 MHz, DMSO-d_6) δ = 207.9, 173.4, 171.9, 168.7, 156.1, 155.8, 130.4, 130.3, 125.8, 125.5, 115.2, 114.7, 94.4, 69.2, 62.3, 40.9, 33.7, 33.4, 27.4, 26.8. The NMR spectra in $CDCl_3$ were not collected due to insolubility. HRMS (ESI-Orbitrap) *m/z* calcd for $C_{12}H_{13}NO_3$ $[M+H]^+$ 220.0968, found 220.0967.

(*S*)-2-(2-(2-Benzyl-3,5-dioxopyrrolidin-1-yl)ethyl)isoindoline-1,3-dione 8a{6,2} **and (*S*)-2-(2-(2-benzyl-3-hydroxy-5-oxo-2,5-dihydro-1*H*-pyrrol-1-yl)ethyl)isoindoline-1,3-dione 8b**{6,2}

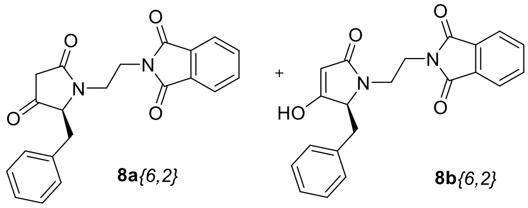

8a{6,2} **8b**{6,2}

Yield 32.3 mg (30 %) of amorphous solid. Mixture of tautomers **8a**{6,2} and **8b**{6,2} in DMSO: ^{1}H NMR (400 MHz, DMSO-d_6) δ = 11.52 (s, 1 H), 7.88 - 7.76 (m, 8 H), 7.30 - 7.05 (m, 10 H), 4.53 (t, *J* = 4.1 Hz, 1 H), 4.44 (s, 1 H), 4.39 (t, *J* = 4.1 Hz, 1 H), 4.09 (ddd, *J* = 4.6, 10.1, 14.2 Hz, 1 H), 4.01 - 3.84 (m, 2 H), 3.80 - 3.65 (m, 2 H), 3.55 (td, *J* = 3.5, 14.1 Hz, 1 H), 3.28 (td, *J* = 3.7, 14.2 Hz, 1 H), 3.21 - 3.03 (m, 4 H), 2.97 (dd, *J* = 3.9, 14.4 Hz, 1 H), 2.66 (d, *J* = 22.0 Hz, 1 H), 2.35 (d, *J* = 21.5 Hz, 1 H). ^{13}C NMR (126 MHz, DMSO-d_6) δ = 206.9, 173.6, 171.8, 169.1, 168.0, 167.6, 135.6, 135.4, 134.3, 134.2, 131.6, 131.5, 129.3, 129.3, 128.3, 127.8, 126.8, 126.3, 123.0, 122.9, 93.8, 66.1, 58.9, 40.5, 37.9, 36.8, 35.8, 34.5, 34.3, 33.5. Only tautomer **8a**{6,2} in $CDCl_3$: ^{1}H NMR (400 MHz, $CDCl_3$) δ = 7.86 - 7.78 (m, 2 H), 7.75 - 7.68 (m, 2 H), 7.29 - 7.22 (m, 3 H, overlap with $CDCl_3$), 7.07 (dd, *J* = 1.4, 7.8 Hz, 2 H), 4.52 (dt, *J* = 1.4, 4.4 Hz, 1 H), 4.45 - 4.36 (m, 1 H), 3.84 (dd, *J* = 3.9, 6.6 Hz, 2 H), 3.21 - 3.06 (m, 3 H), 2.74 - 2.62 (m, 1 H), 2.34 - 2.22 (m, 1 H). ^{13}C NMR (101 MHz, $CDCl_3$) δ = 205.9, 169.8, 168.4, 134.7, 134.2, 131.8, 129.3, 128.9, 127.4, 123.4, 66.9, 40.7, 38.9, 35.3, 34.7. HRMS (ESI-Orbitrap) *m/z* calcd for $C_{21}H_{18}N_2O_4$ $[M+H]^+$ 363.1339, found 363.1338.

(*S*)-5-Benzyl-1-(prop-2-yn-1-yl)pyrrolidine-2,4-dione 8a*{6,4}* and (*S*)-5-benzyl-4-hydroxy-1-(prop-2-yn-1-yl)-1,5-dihydro-2*H*-pyrrol-2-one 8b*{6,4}*

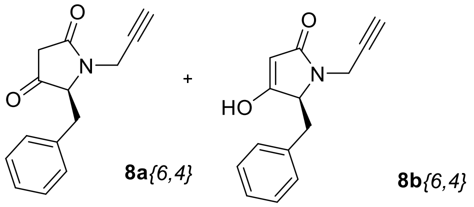

8a*{6,4}* **8b***{6,4}*

Yield 18.8 mg (21 %) of amorphous solid. Mixture of tautomers **8a***{7,4}* and **8b***{7,4}* in DMSO: ^{1}H NMR (400 MHz, DMSO-d_6) δ = 11.75 (s, 1 H), 7.32 - 7.11 (m, 10 H), 4.66 (s, 1 H), 4.56 (dd, *J* = 2.5, 17.6 Hz, 1 H), 4.40 - 4.31 (m, 2 H), 4.25 (t, *J* = 4.4 Hz, 1 H), 4.01 (dd, *J* = 2.1, 17.2 Hz, 1 H), 3.75 (dd, *J* = 2.5, 17.6 Hz, 1 H), 3.32 (t, *J* = 2.5 Hz, 1 H), 3.22 - 3.14 (m, 3 H), 3.11 (dd, *J* = 2.1, 4.8 Hz, 1 H), 3.03 - 2.92 (m, 2 H). ^{13}C NMR (101 MHz, DMSO-d_6) δ = 206.3, 174.5, 172.1, 168.5, 135.6, 135.6, 129.5, 128.4, 127.9, 126.8, 126.4, 94.0, 79.7, 78.1, 75.3, 74.1, 66.7, 59.7, 40.9, 34.4, 34.0, 29.5, 29.1. Only tautomer **8a***{6,4}* in $CDCl_3$: ^{1}H NMR (400 MHz, $CDCl_3$) δ = 7.35 - 7.22 (m, 3 H, overlap with $CDCl_3$), 7.13 - 7.03 (m, 2 H), 4.91 (dd, *J* = 2.3, 17.9 Hz, 1 H), 4.46 (dt, *J* = 1.4, 4.4 Hz, 1 H), 3.82 (d, *J* = 17.4 Hz, 1 H), 3.25 - 3.12 (m, 2 H), 2.83 (d, *J* = 22.4 Hz, 1 H), 2.35 (t, *J* = 2.5 Hz, 1 H), 2.35 (d, *J* = 22.4 Hz, 1 H). ^{13}C NMR (101 MHz, $CDCl_3$) δ = 205.6, 168.4, 134.2, 129.5, 128.9, 127.5, 76.3, 73.7, 66.9, 41.2, 35.3, 30.0. HRMS (ESI-Orbitrap) *m/z* calcd for $C_{14}H_{13}NO_2$ $[M+H]^+$ 228.1019, found 228.1019.

(*S*)-2-(2-(2-(Hydroxymethyl)-3,5-dioxopyrrolidin-1-yl)ethyl)isoindoline-1,3-dione 8a*{7,2}* and (*S*)-2-(2-(3-hydroxy-2-(hydroxymethyl)-5-oxo-2,5-dihydro-1*H*-pyrrol-1-yl)ethyl)iso-indoline-1,3-dione 8b*{7,2}*

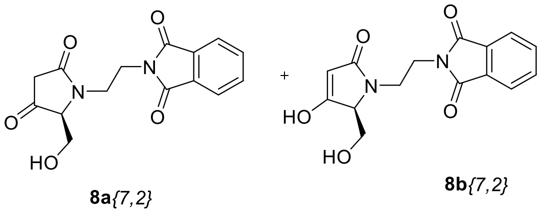

8a*{7,2}* **8b***{7,2}*

Yield 34.7 mg (52 %) of amorphous solid. Mixture of tautomers **8a***{7,2}* and **8b***{7,2}* in DMSO: ^{1}H NMR (400 MHz, DMSO-d_6) δ = 11.44 (br. s., 1 H), 7.90 - 7.77 (m, 8 H), 5.15 (br. s., 1 H), 4.61 (s, 1 H), 4.17 (br. s., 1 H), 4.11 - 3.97 (m, 2 H), 3.94 - 3.77 (m, 4 H), 3.76 - 3.62 (m, 4 H), 3.34 - 3.18 (m, 3 H), 2.87 (d, *J* = 22.0 Hz, 1 H), 2.79 (d, *J* = 22.0 Hz, 1 H). ^{13}C NMR (101 MHz, DMSO-d_6) δ = 207.2, 172.5, 172.4, 169.8, 168.1, 167.8, 134.4, 134.3, 131.7, 131.6, 123.1, 123.0, 93.9, 68.1, 61.8, 59.2, 58.1, 41.3, 37.9, 37.5, 36.4, 34.7. Only tautomer **8a***{7,2}* in $CDCl_3$: ^{1}H NMR (400 MHz, $CDCl_3$) δ = 7.82 - 7.70 (m, 2 H), 7.69 - 7.58 (m, 2 H), 5.30 - 4.59 (m, 1 H), 4.07 (br. s., 1 H), 4.02 - 3.93 (m, 1 H), 3.91 (br. s., 2 H), 3.87 - 3.79 (m, 2 H), 3.51 (td, *J* = 4.5,

14.3 Hz, 1 H), 2.91 - 2.79 (m, 1 H), 2.79 - 2.69 (m, 1 H). ^{13}C NMR (101 MHz, $CDCl_3$) δ = 205.3, 170.4, 168.4, 134.0, 131.7, 123.2, 69.3, 59.3, 41.3, 39.5, 35.5. HRMS (ESI-Orbitrap) *m/z* calcd for $C_{15}H_{14}N_2O_5$ $[M+H]^+$ 303.0975, found 303.0976.

(*S*)-3-(1-(2-(1,3-Dioxoisoindolin-2-yl)ethyl)-3,5-dioxopyrrolidin-2-yl)propanoic acid 8a*{8,2}* **and (*S*)-3-(1-(2-(1,3-dioxoisoindolin-2-yl)ethyl)-3-hydroxy-5-oxo-2,5-dihydro-1*H*-pyrrol-2-yl)propanoic acid 8b***{8,2}*

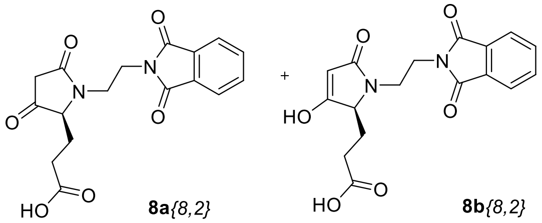

8a*{8,2}* **8b***{8,2}*

Yield 14.8 mg (21 %) of amorphous solid. Mixture of tautomers **8a***{8,2}* and **8b***{8,2}* in DMSO: ^{1}H NMR (400 MHz, DMSO-d_6) δ = 12.22 (br. s., 1 H), 11.50 (br. s., 1 H), 7.90 - 7.76 (m, 8 H), 4.64 (s, 1 H), 4.29 - 4.21 (m, 1 H), 4.19 (s, 1 H), 3.99 (ddd, *J* = 4.4, 9.5, 14.1 Hz, 1 H), 3.93 - 3.80 (m, 2 H), 3.79 - 3.65 (m, 3 H), 3.58 (td, *J* = 3.9, 13.7 Hz, 2 H), 3.21 (td, *J* = 3.9, 14.2 Hz, 1 H), 3.13 (d, *J* = 22.0 Hz, 1 H), 3.10 - 3.04 (m, 1 H), 2.79 (d, *J* = 22.0 Hz, 1 H), 2.36 - 2.12 (m, 2 H), 2.11 - 1.91 (m, 5 H). ^{13}C NMR (101 MHz, DMSO-d_6) δ = 207.8, 174.0, 173.9, 173.6, 172.1, 169.6, 168.1, 167.8, 134.4, 134.3, 131.7, 131.6, 123.1, 123.0, 93.6, 64.4, 57.7, 40.8, 37.9, 36.7, 36.1, 34.7, 28.4, 27.0, 23.8, 22.6. Only tautomer **8a***{8,2}* in $CDCl_3$: ^{1}H NMR (400 MHz, $CDCl_3$) δ = 10.05 (br. s., 1 H), 7.86 - 7.76 (m, 2 H), 7.75 - 7.65 (m, 2 H), 4.40 - 4.22 (m, 2 H), 3.99 - 3.80 (m, 2 H), 3.18 (td, *J* = 3.3, 14.5 Hz, 1 H), 2.91 (d, *J* = 22.0 Hz, 1 H), 2.84 (d, *J* = 22.0 Hz, 1 H), 2.43 - 2.31 (m, 2 H), 2.23 (dtd, *J* = 2.7, 7.4, 14.5 Hz, 1 H), 2.07 - 1.93 (m, 1 H). ^{13}C NMR (101 MHz, $CDCl_3$) δ = 206.0, 174.6, 169.9, 168.4, 134.1, 131.8, 123.3, 64.5, 40.8, 38.6, 34.7, 28.4, 23.7. HRMS (ESI-Orbitrap) *m/z* calcd for $C_{17}H_{16}N_2O_6$ $[M+H]^+$ 345.1081, found 345.1081.

Ammonium 1,1'-dimethyl-2,5'-dioxo-2,2',5,5'-tetrahydro-1*H*,1'*H*-[3,3'-bipyrrol]-4-olate 9b*{1,1}*

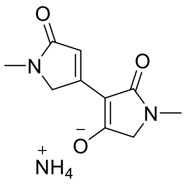

Crude product was purified in MeCN/10 mM aqueous ammonium acetate and left overnight at room temperature for quantitative self-condensation: 9.6 mg (47 %) of amorphous solid. ^{1}H NMR (400 MHz, DMSO-d_6) δ = 6.26 (s, 1 H), 4.27 (s, 2 H), 3.95 (s, 2 H), 2.88 (s, 3 H), 2.82 (s, 3

H). ^{13}C NMR (101 MHz, DMSO-d_6) δ = 171.5, 171.4, 169.8, 146.7, 115.9, 99.1, 54.8, 51.4, 28.3, 28.1. HRMS (ESI-Orbitrap) *m/z* calcd for $C_{10}H_{12}N_2O_3$ $[M+H]^+$ 209.0921, found 209.0921.

Ammonium 1,1'-bis(2-(1,3-dioxoisoindolin-2-yl)ethyl)-2,5'-dioxo-2,2',5,5'-tetrahydro-1*H*,1'*H*-[3,3'-bipyrrol]-4-olate 9b*{1,2}*

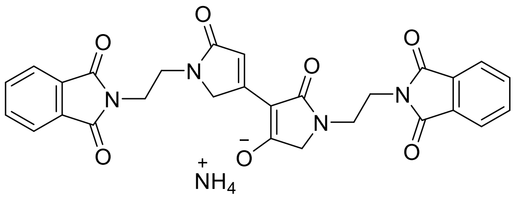

Crude product was purified in MeCN/10 mM aqueous ammonium acetate: 27.4 mg (53 %), and by self-condensation of **8***{1,2}* exposed to 10 mM aqueous ammonium acetate for 24 h at room temperature and purified by MeCN/10 mM aqueous ammonium acetate: 10.9 mg (80%) of amorphous solid. ^1H NMR (400 MHz, DMSO-d_6) δ = 7.87 - 7.78 (m, 8 H), 5.90 (s, 1 H), 4.21 (s, 2 H), 4.04 (br. s., 2 H), 3.78 - 3.71 (m, 2 H), 3.71 - 3.65 (m, 2 H), 3.61 - 3.51 (m, 4 H). ^{13}C NMR (101 MHz, DMSO-d_6) δ = 171.8, 171.3, 169.9, 167.8, 167.7, 146.9, 134.3, 134.3, 131.6, 131.6, 123.1, 123.0, 115.5, 52.4, 49.5, 36.1, 35.9. HRMS (ESI-Orbitrap) *m/z* calcd for $C_{28}H_{22}N_4O_7$ $[M+H]^+$ 527.1561, found 527.1564.

Ammonium (6*S*,6'*R*,7aS,7'a*S*)-6,6'-bis(benzyloxy)-3,3'-dioxo-5,5',6,6',7,7a,7',7'a-octahydro-3*H*,3'*H*-[1,2'-bipyrrolizin]-1'-olate 9b*{2,-}*

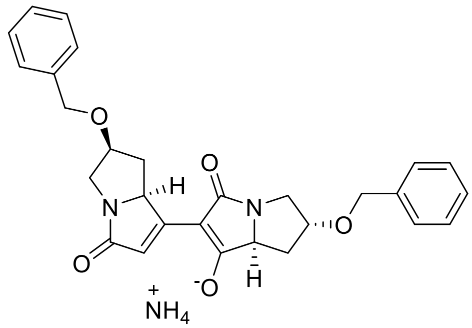

Crude product was purified in MeCN/10 mM aqueous ammonium acetate: 31.9 mg (37 %) of amorphous solid. ^1H NMR (400 MHz, DMSO-d_6) δ = 7.39 - 7.25 (m, 10 H), 6.00 (s, 1 H), 4.77 (dd, *J* = 6.0, 10.1 Hz, 1 H), 4.56 - 4.44 (m, 5 H), 4.36 (t, *J* = 5.0 Hz, 1 H), 4.29 (t, *J* = 5.0 Hz, 1 H), 4.07 (dd, *J* = 6.6, 9.8 Hz, 1 H), 3.67 (dd, *J* = 5.5, 11.9 Hz, 1 H), 3.59 (dd, *J* = 5.5, 12.4 Hz, 1 H), 3.03 (dd, *J* = 12.6, 16.7 Hz, 2 H), 2.67 (dd, *J* = 5.7, 13.1 Hz, 1 H), 2.24 (dd, *J* = 6.4, 12.8 Hz, 1 H), 1.47 (ddd, *J* = 5.0, 10.1, 12.8 Hz, 1 H), 1.17 (ddd, *J* = 5.0, 10.4, 12.9 Hz, 1 H). ^{13}C NMR (101 MHz, DMSO-d_6) δ = 184.3, 177.8, 176.9, 156.0, 138.4, 128.3, 127.6, 127.4, 110.2, 95.8, 82.6, 81.2, 70.1, 70.0, 65.7, 63.3, 50.1, 49.5, 36.3, 34.6. HRMS (ESI-Orbitrap) *m/z* calcd for $C_{28}H_{28}N_2O_5$ $[M+H]^+$ 473.2071, found 473.2074.

Ammonium (2'*S*,5*S*)-1,1'-dibenzyl-2',5-dimethyl-2,5'-dioxo-2,2',5,5'-tetrahydro-1*H*,1'*H*-[3,3'-bipyrrol]-4-olate 9b*{5,3}*

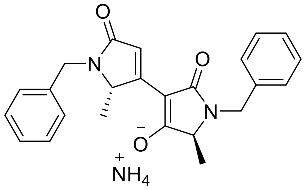

Crude product was purified in MeCN/10 mM aqueous ammonium acetate: 7.9 mg (22 %) of amorphous solid. ^{1}H NMR (400 MHz, DMSO-*d$_6$*) δ = 7.37 - 7.26 (m, 5 H), 7.26 - 7.17 (m, 5 H), 6.27 (s, 1 H), 4.91 (d, *J* = 15.6 Hz, 1 H), 4.77 (d, *J* = 15.6 Hz, 1 H), 4.39 (q, *J* = 6.4 Hz, 1 H), 4.16 (d, *J* = 15.6 Hz, 1 H), 4.14 (d, *J* = 15.6 Hz, 1 H), 3.68 (q, *J* = 6.4 Hz, 1 H), 1.22 (d, *J* = 6.4 Hz, 3 H), 1.19 (d, *J* = 6.4 Hz, 3 H). ^{13}C NMR (101 MHz, DMSO-*d$_6$*) δ = 178.4, 170.7, 169.7, 154.2, 138.6, 138.5, 128.53, 128.50, 127.54, 127.48 127.03, 126.99, 113.8, 67.8, 56.9, 55.2, 42.5, 42.3, 17.7, 16.2. HRMS (ESI-Orbitrap) *m/z* calcd for $C_{24}H_{24}N_2O_3$ [M+H]$^+$ 389.1860, found 389.1861.

Ammonium (2'*S*,5*S*)-2',5-dibenzyl-1,1'-bis(2-(1,3-dioxoisoindolin-2-yl)ethyl)-2,5'-dioxo-2,2',5,5'-tetrahydro-1*H*,1'*H*-[3,3'-bipyrrol]-4-olate 9b*{6,2}*

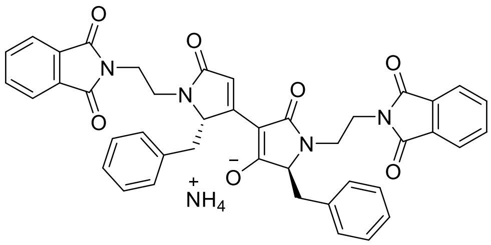

Crude product was purified in MeCN/10 mM aqueous ammonium acetate: 3.9 mg (49 %) of amorphous solid and by spontaneous self-condensation of **8***{6,2}* and purified by MeCN/10 mM aqueous ammonium acetate: 6.1 mg (53%). ^{1}H NMR (400 MHz, DMSO-*d$_6$*) δ = 7.87 - 7.77 (m, 8 H), 7.28 - 7.20 (m, 2 H), 7.19 - 7.10 (m, 3 H), 7.08 - 7.01 (m, 1 H), 7.01 - 6.94 (m, 2 H), 6.63 (d, *J* = 7.3 Hz, 2 H), 5.66 (s, 1 H), 4.82 (t, *J* = 3.9 Hz, 1 H), 4.63 (t, *J* = 3.7 Hz, 1 H), 4.00 (ddd, *J* = 4.6, 9.4, 14.4 Hz, 1 H), 3.86 - 3.71 (m, 2 H), 3.70 - 3.52 (m, 2 H), 3.45 (d, *J* = 4.6 Hz, 2 H, overlap with the water), 3.33 - 3.25 (m, 1 H, overlap with the water), 3.23 - 3.08 (m, 2 H), 2.97 (dd, *J* = 4.1, 14.2 Hz, 1 H), 2.63 (dd, *J* = 4.1, 14.2 Hz, 1 H). ^{13}C NMR (101 MHz, DMSO-*d$_6$*) δ = 173.0, 170.8, 169.3, 167.9, 167.5, 149.9, 135.8, 135.1, 134.4, 134.3, 131.6, 129.2, 129.1, 128.1, 127.7, 126.7, 126.0, 123.1, 122.9, 117.8, 99.8, 60.6, 58.6, 37.6, 37.3, 36.0, 35.8, 35.4, 33.8. HRMS (ESI-Orbitrap) *m/z* calcd for $C_{42}H_{34}N_4O_7$ [M-H]$^-$ 705.2344, found 705.2349.

^{1}H and ^{13}C NMR spectra (d_6-DMSO) of 2-(2-(2,4-dioxopyrrolidin-1-yl)ethyl)isoindoline-1,3-dione 8a*{1,2}* and 2-(2-(4-hydroxy-2-oxo-2,5-dihydro-1H-pyrrol-1-yl)ethyl)isoindoline-1,3-dione 8b*{1,2}*

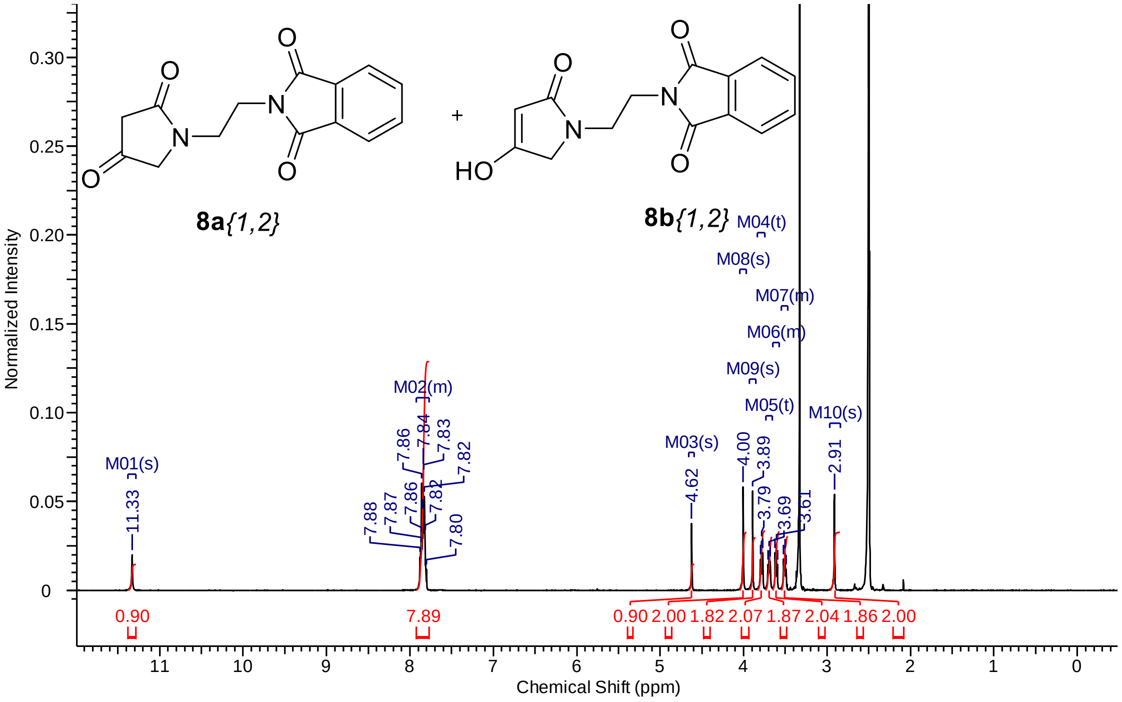

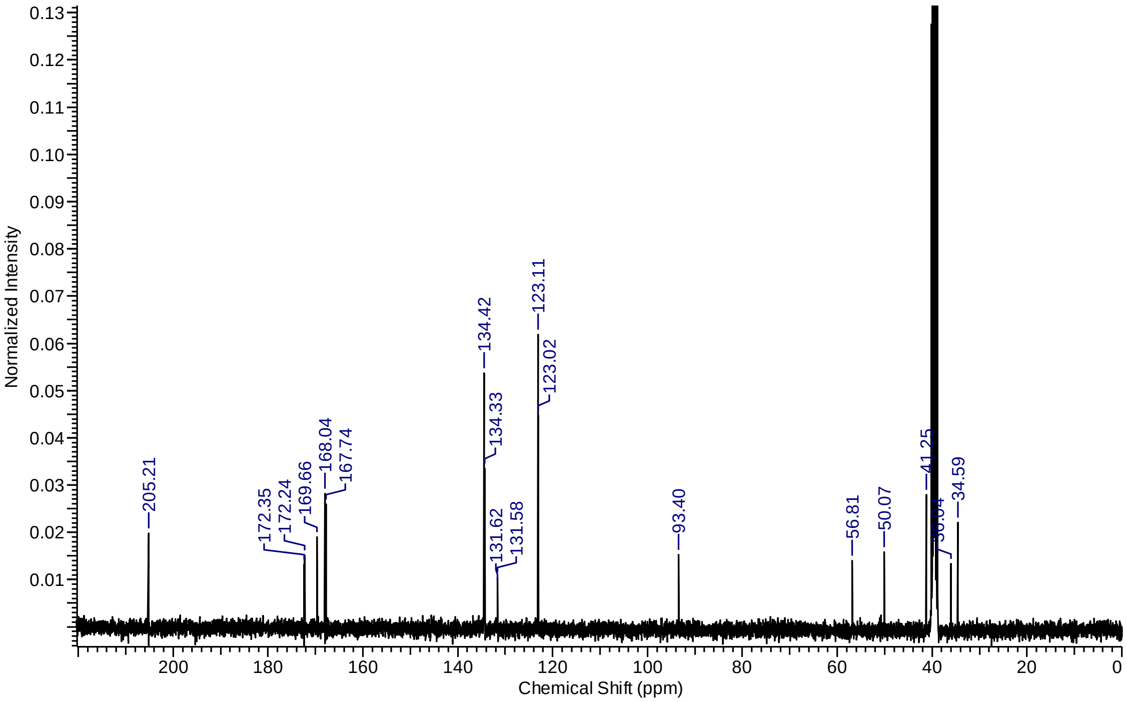

^{1}H and ^{13}C NMR spectra ($CDCl_3$) of 2-(2-(2,4-dioxopyrrolidin-1-yl)ethyl)isoindoline-1,3-dione 8a*{1,2}*

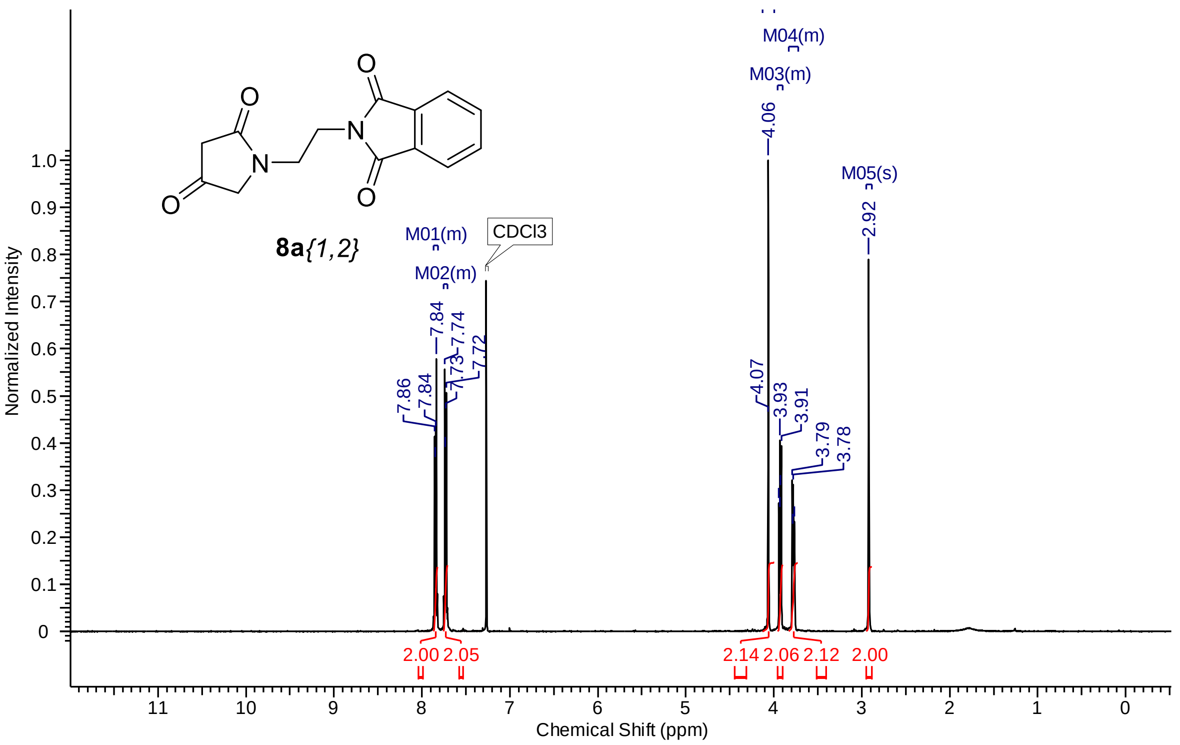

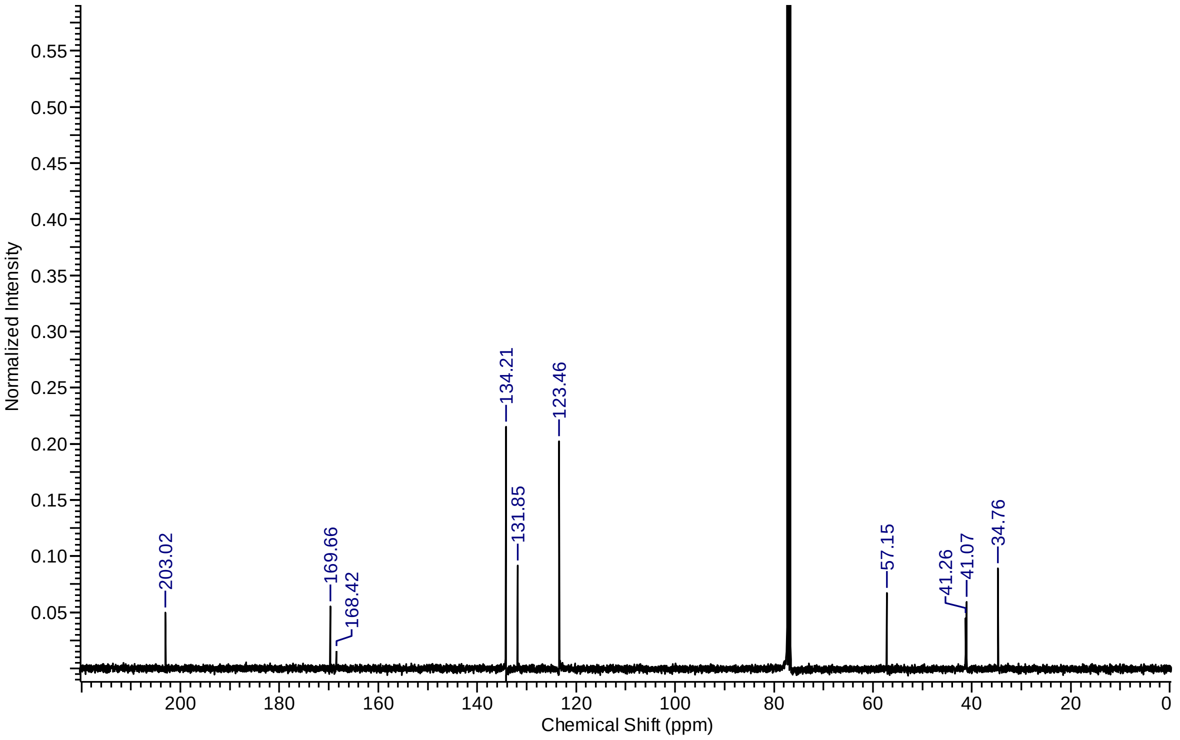

^{1}H and ^{13}C NMR spectra (d_6-DMSO) of (*S*)-9,9a-dihydro-1*H*-pyrrolo[1,2-*a*]indole-1,3(2*H*)-dione 8a*{3,-}* and (*S*)-1-hydroxy-9,9a-dihydro-3*H*-pyrrolo[1,2-*a*]indol-3-one and 8b*{3,-}*

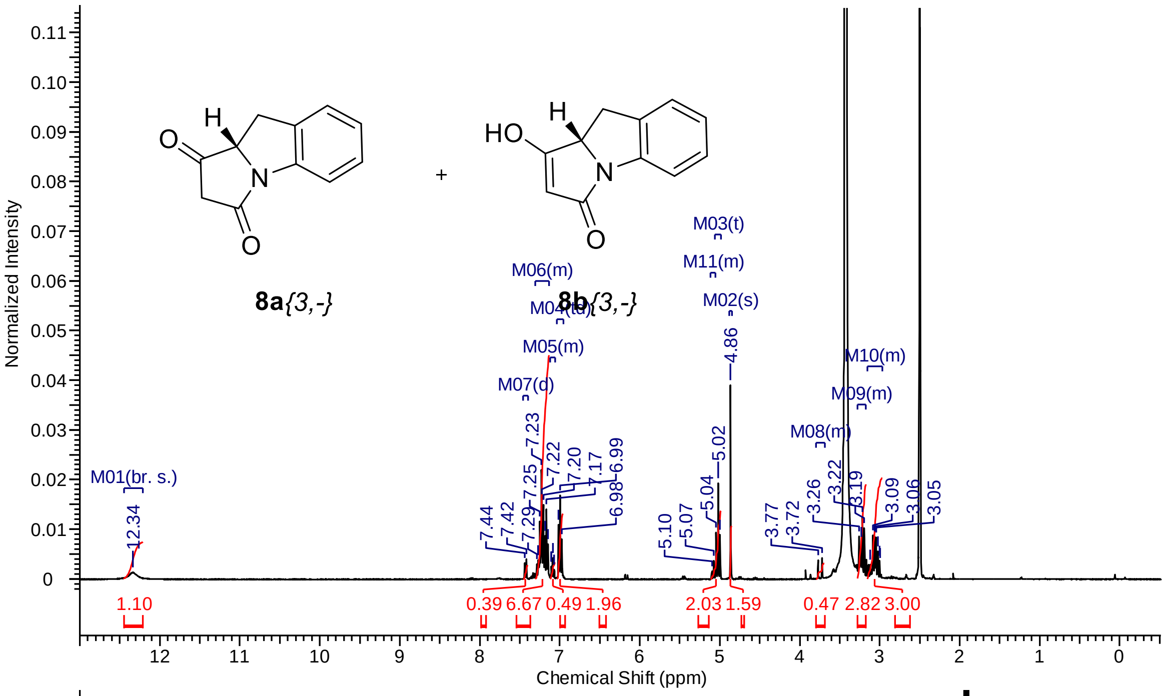

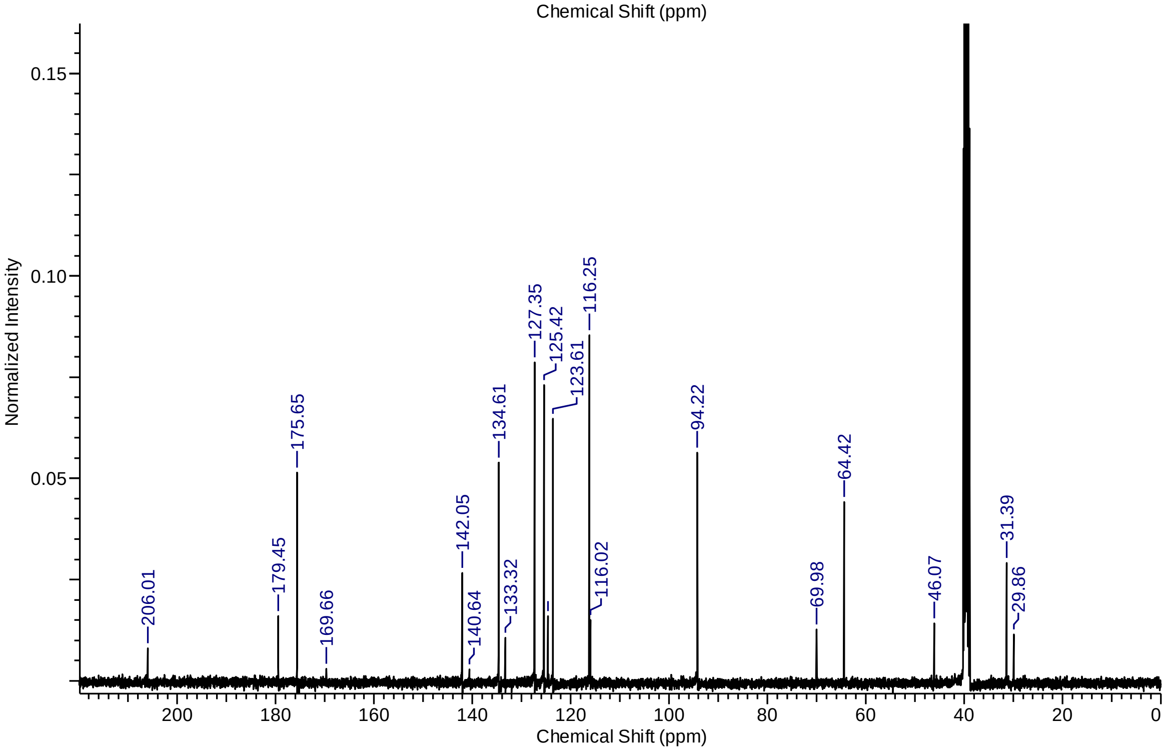

^{1}H and ^{13}C NMR spectra (d_6-DMSO) of (*S*)-5-(4-hydroxybenzyl)-1-methylpyrrolidine-2,4-dione and 8a*{4,1}* and (*S)*-4-hydroxy-5-(4-hydroxybenzyl)-1-methyl-1,5-dihydro-2*H*-pyrrol-2-one 8b*{4,1}*

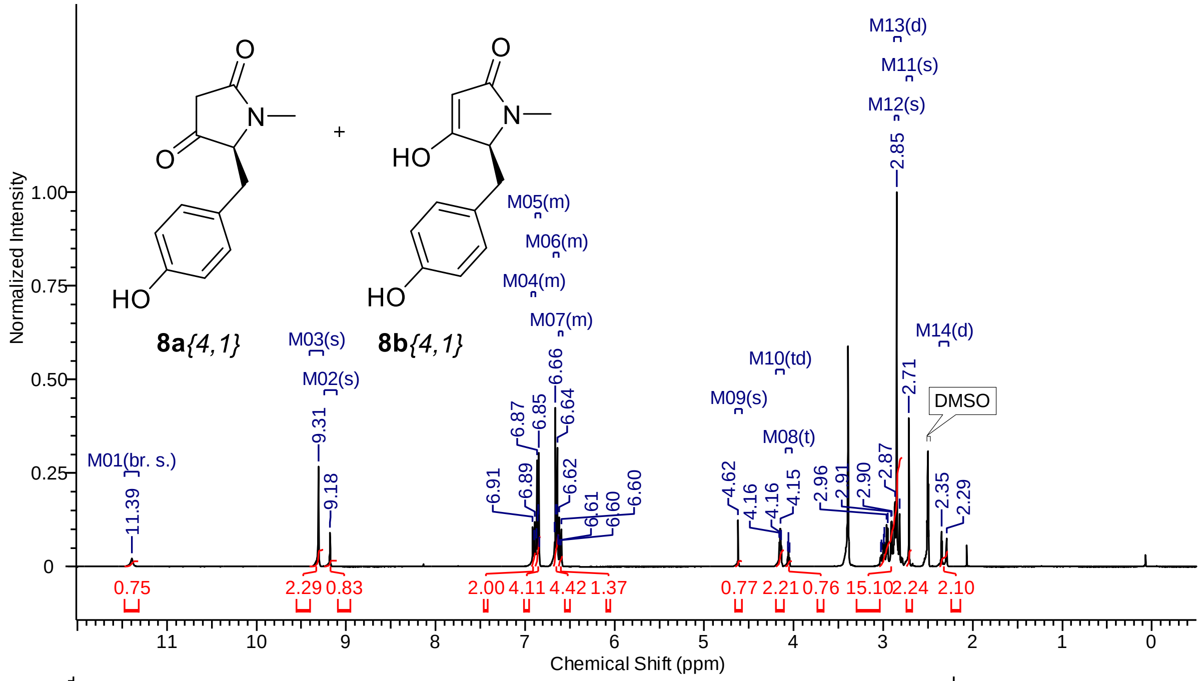

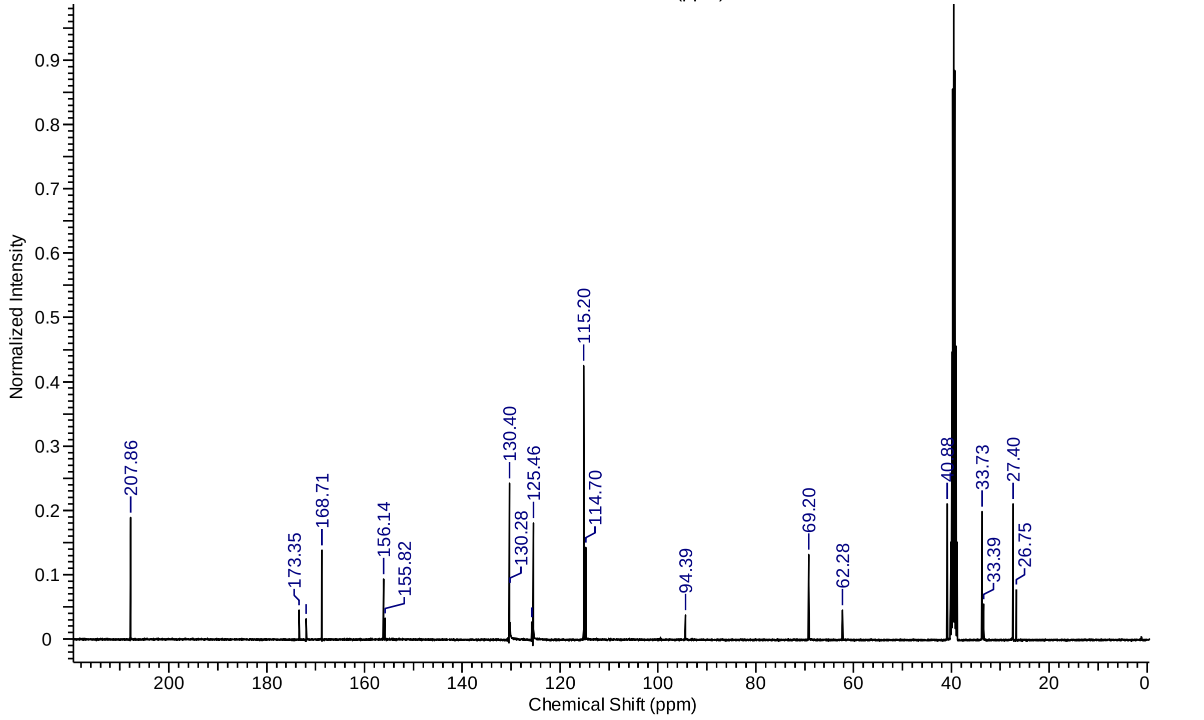

^{1}H and ^{13}C NMR spectra (d_6-DMSO) of (*S*)-2-(2-(2-benzyl-3,5-dioxopyrrolidin-1-yl)ethyl)isoindoline-1,3-dione 8a*{6,2}* and (*S*)-2-(2-(2-benzyl-3-hydroxy-5-oxo-2,5-dihydro-1*H*-pyrrol-1-yl)ethyl)isoindoline-1,3-dione 8b*{6,2}*

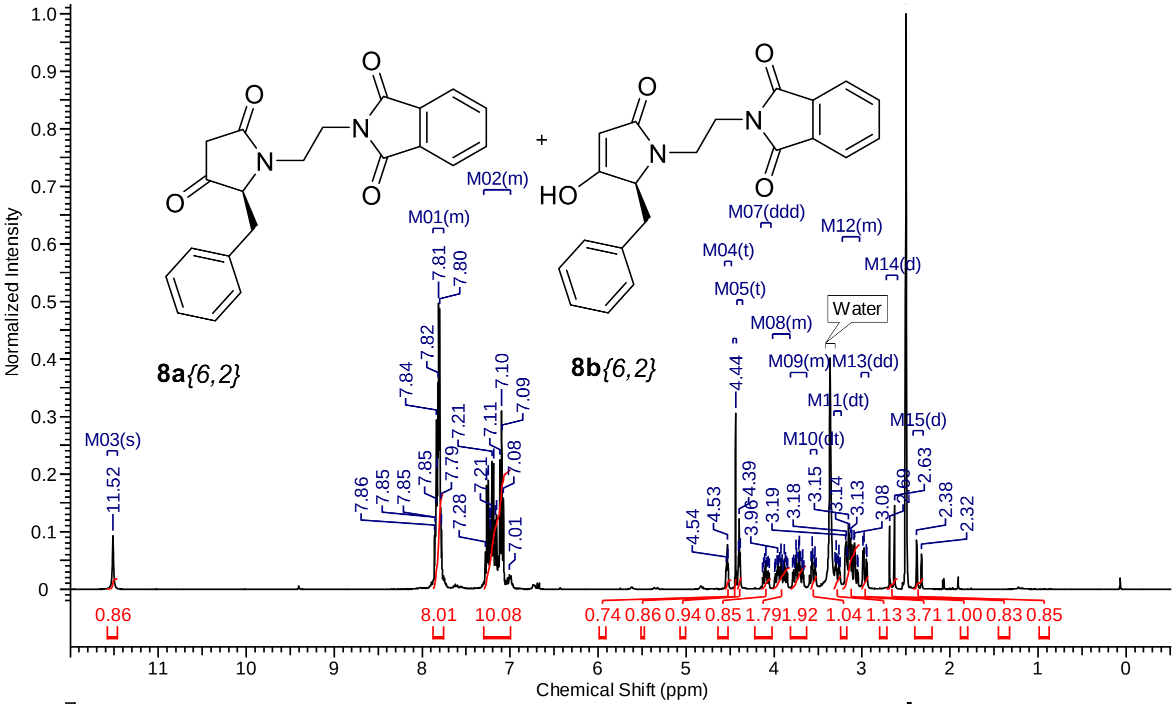

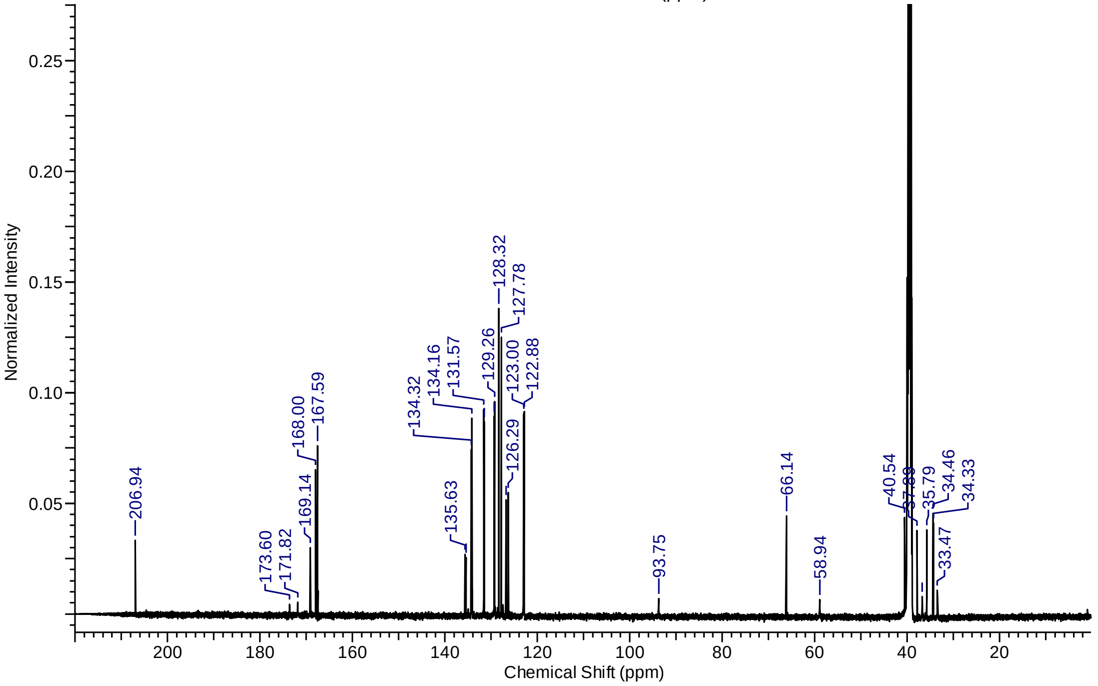

^{1}H and ^{13}C NMR spectra ($CDCl_3$) of (*S*)-2-(2-(2-benzyl-3,5-dioxopyrrolidin-1-yl)ethyl)isoindoline-1,3-dione 8a*{6,2}*

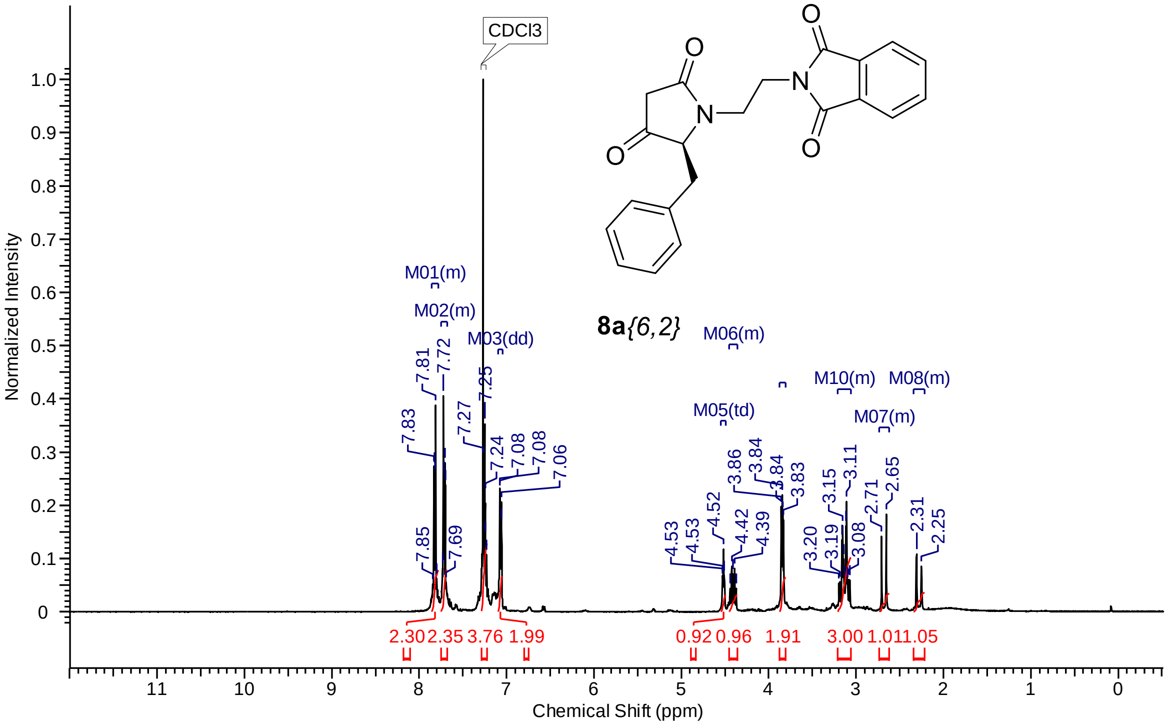

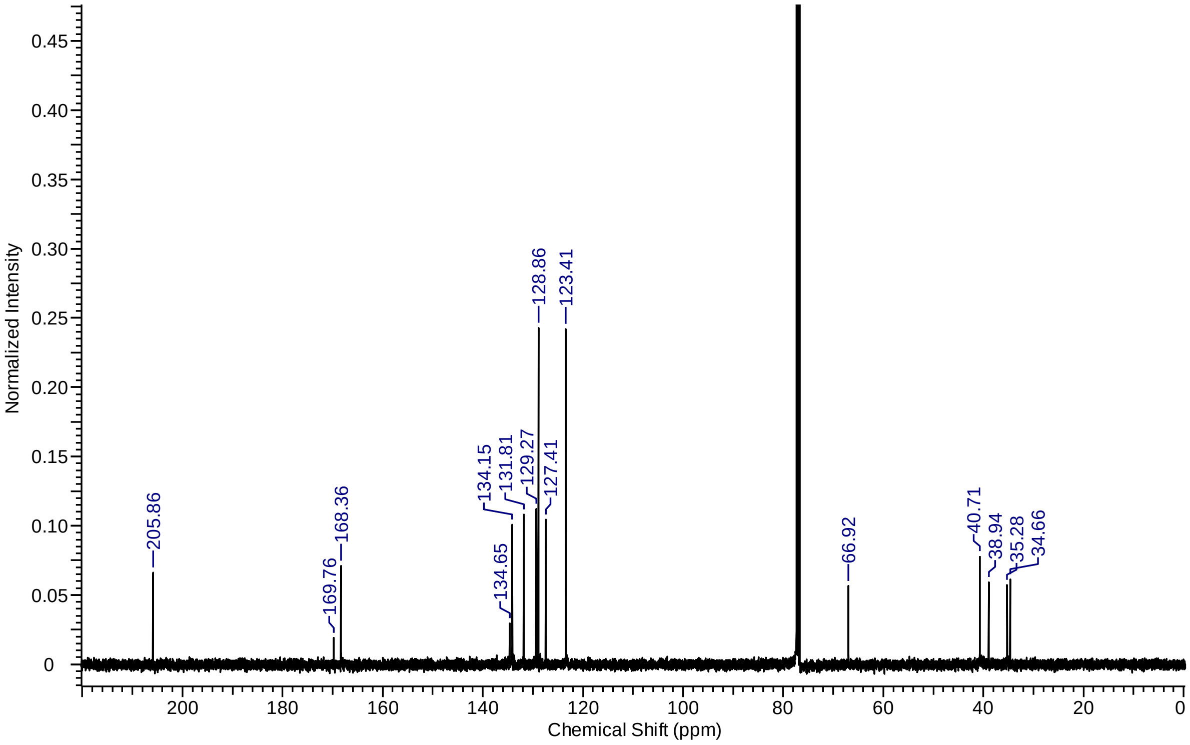

^{1}H and ^{13}C NMR spectra (d_6-DMSO) of (*S*)-5-benzyl-1-(prop-2-yn-1-yl)pyrrolidine-2,4-dione 8a*{6,4}* and (*S*)-5-benzyl-4-hydroxy-1-(prop-2-yn-1-yl)-1,5-dihydro-2*H*-pyrrol-2-one 8b*{6,4}*

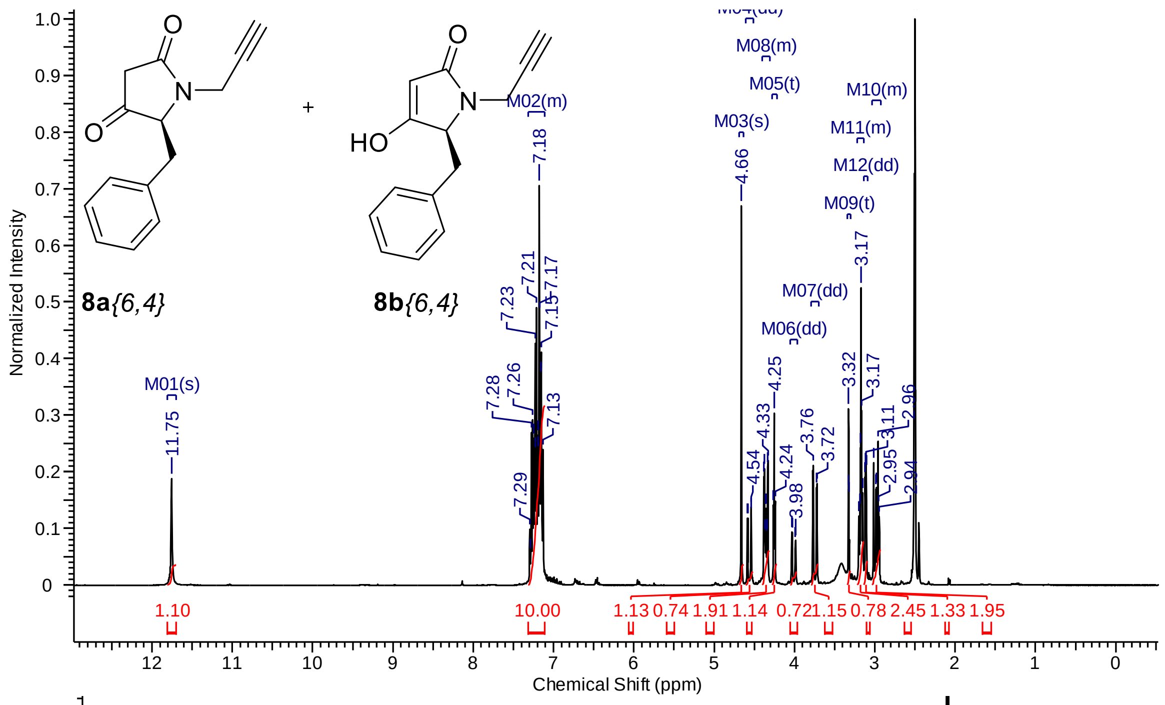

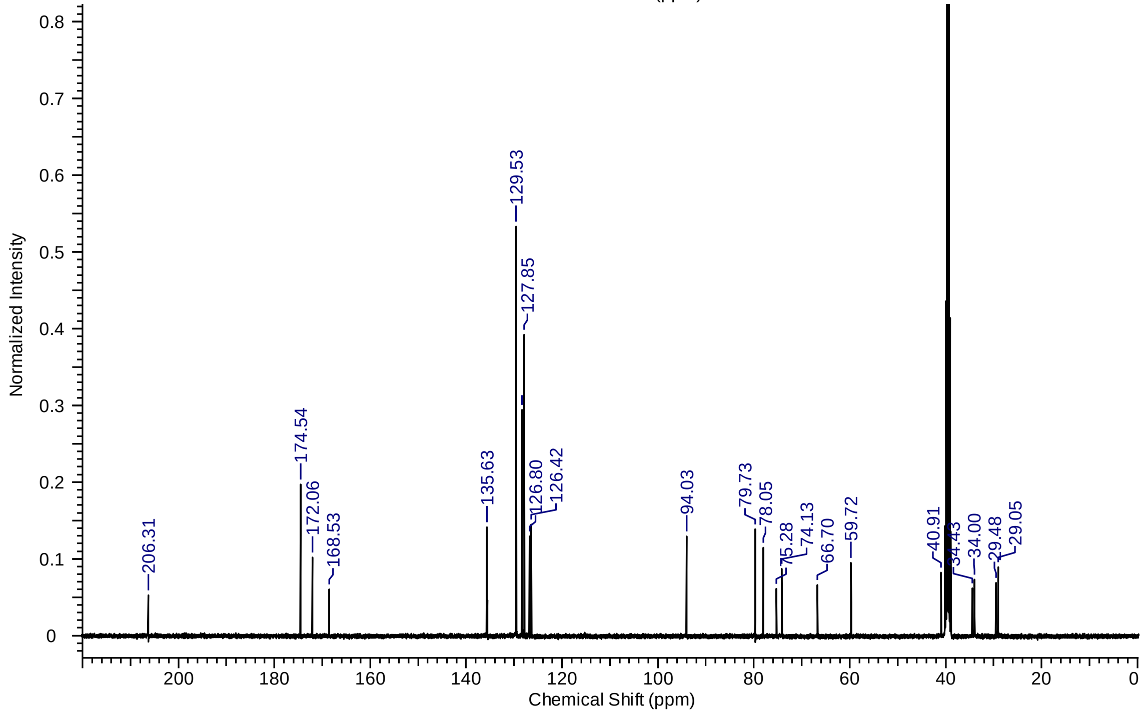

^{1}H and ^{13}C NMR spectra ($CDCl_3$) of (*S*)-5-benzyl-1-(prop-2-yn-1-yl)pyrrolidine-2,4-dione

8a*{6,4}*

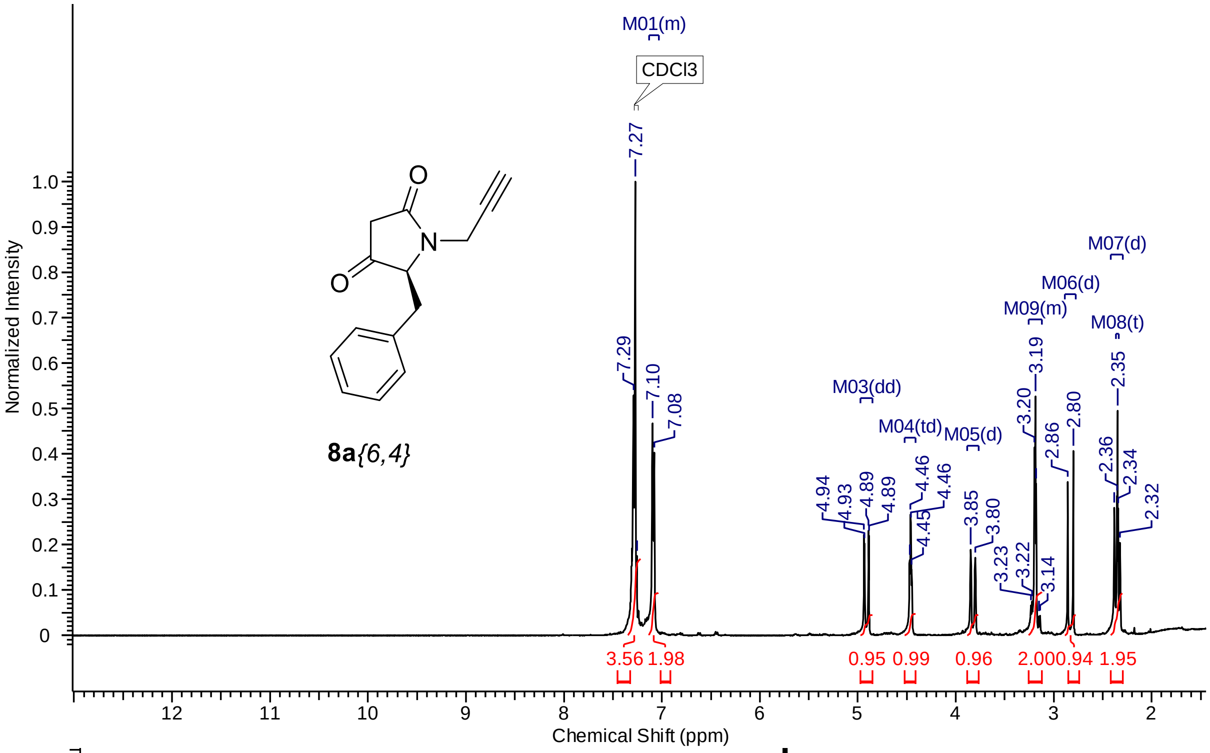

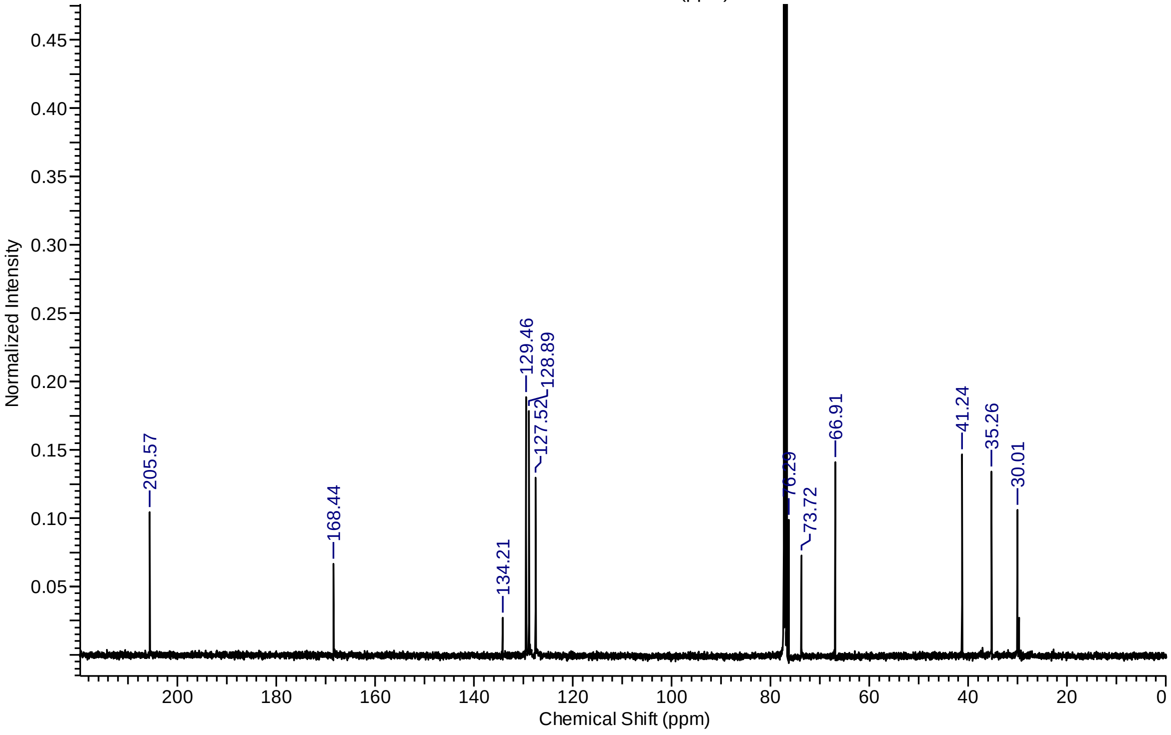

^{1}H and ^{13}C NMR spectra (d_6-DMSO) of (*S*)-2-(2-(2-(hydroxymethyl)-3,5-dioxopyrrolidin-1-yl)ethyl)isoindoline-1,3-dione 8a*{7,2}* and (*S*)-2-(2-(3-hydroxy-2-(hydroxymethyl)-5-oxo-2,5-dihydro-1*H*-pyrrol-1-yl)ethyl)isoindoline-1,3-dione 8b*{7,2}*

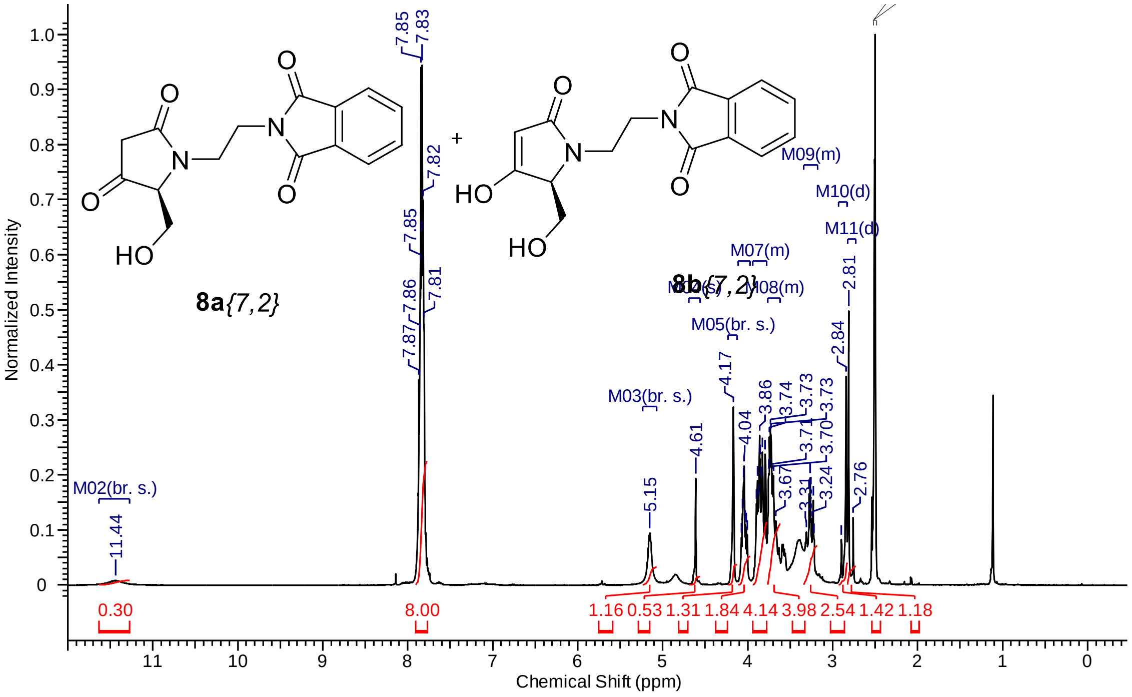

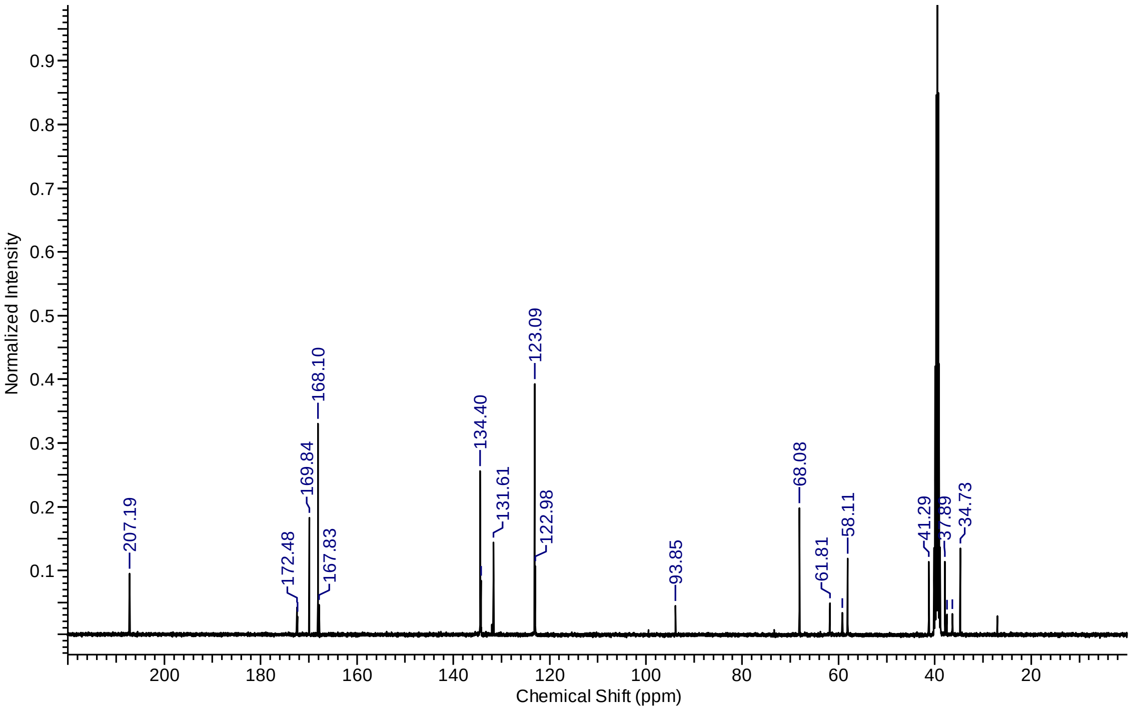

^{1}H and ^{13}C NMR spectra ($CDCl_3$) of (*S*)-2-(2-(2-(hydroxymethyl)-3,5-dioxopyrrolidin-1-yl)ethyl)isoindoline-1,3-dione 8a*{7,2}*

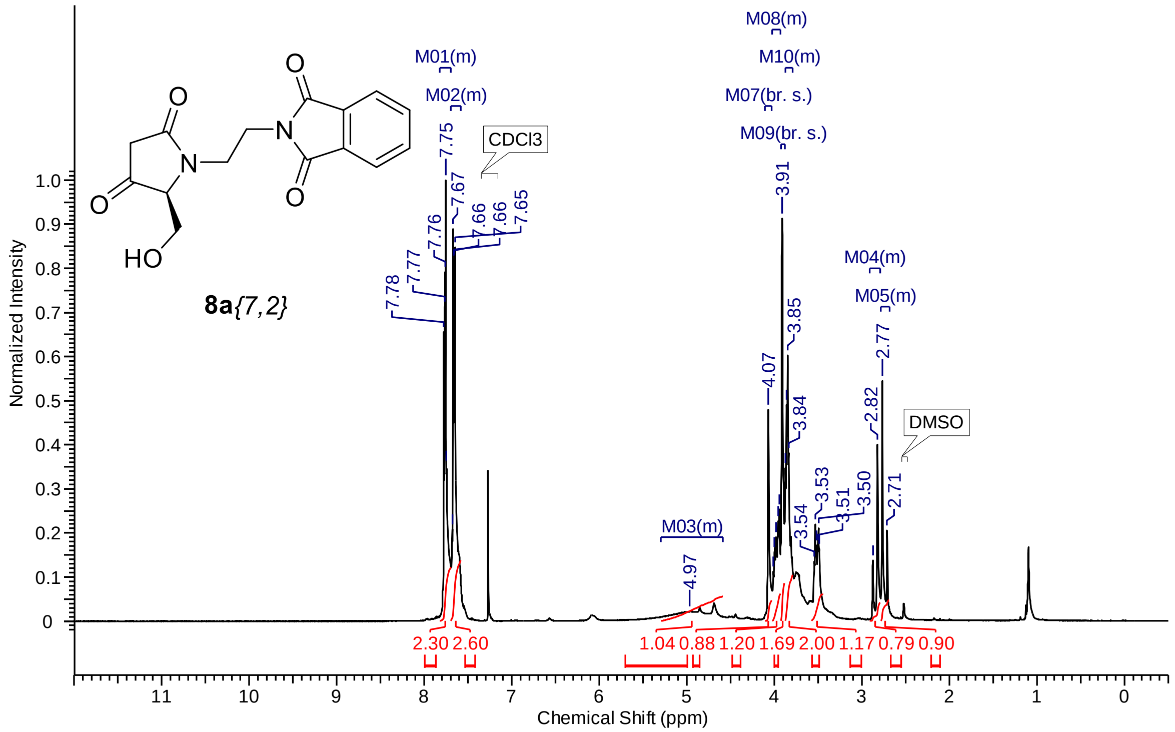

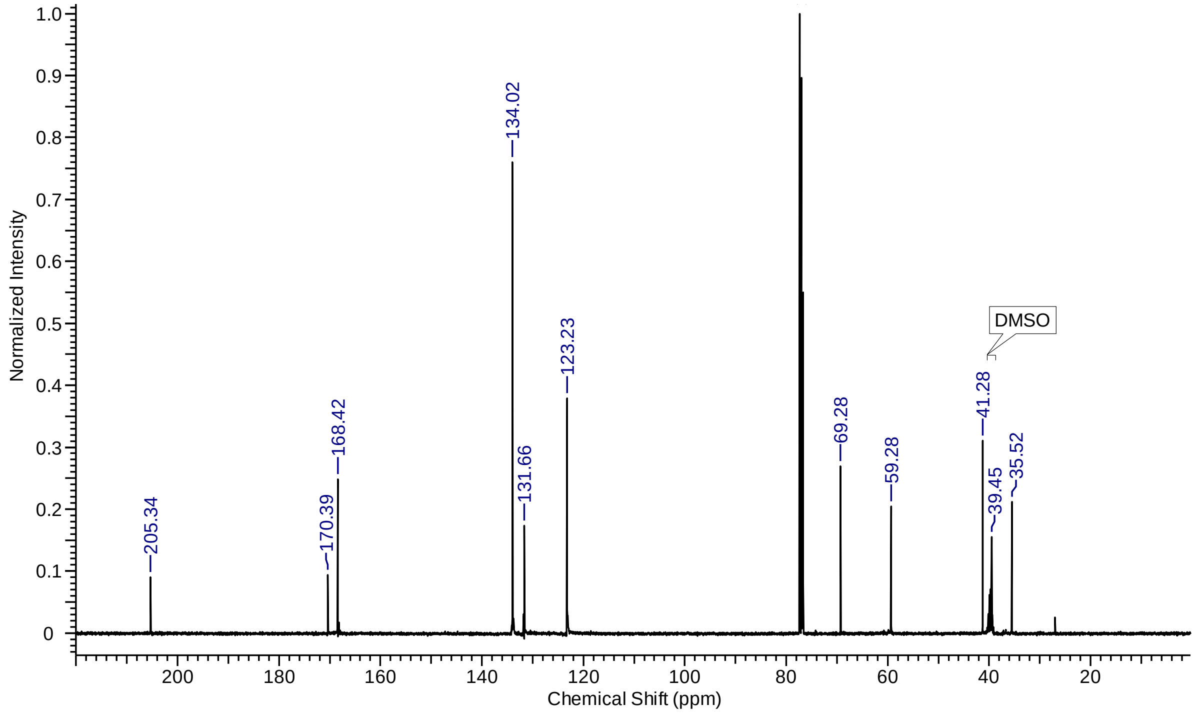

^{1}H and ^{13}C NMR spectra (*d*$_6$-DMSO) of (*S*)-3-(1-(2-(1,3-dioxoisoindolin-2-yl)ethyl)-3,5-dioxopyrrolidin-2-yl)propanoic acid 8a*{8,2}* and (*S*)-3-(1-(2-(1,3-dioxoisoindolin-2-yl)ethyl)-3-hydroxy-5-oxo-2,5-dihydro-1*H*-pyrrol-2-yl)propanoic acid 8b*{8,2}*

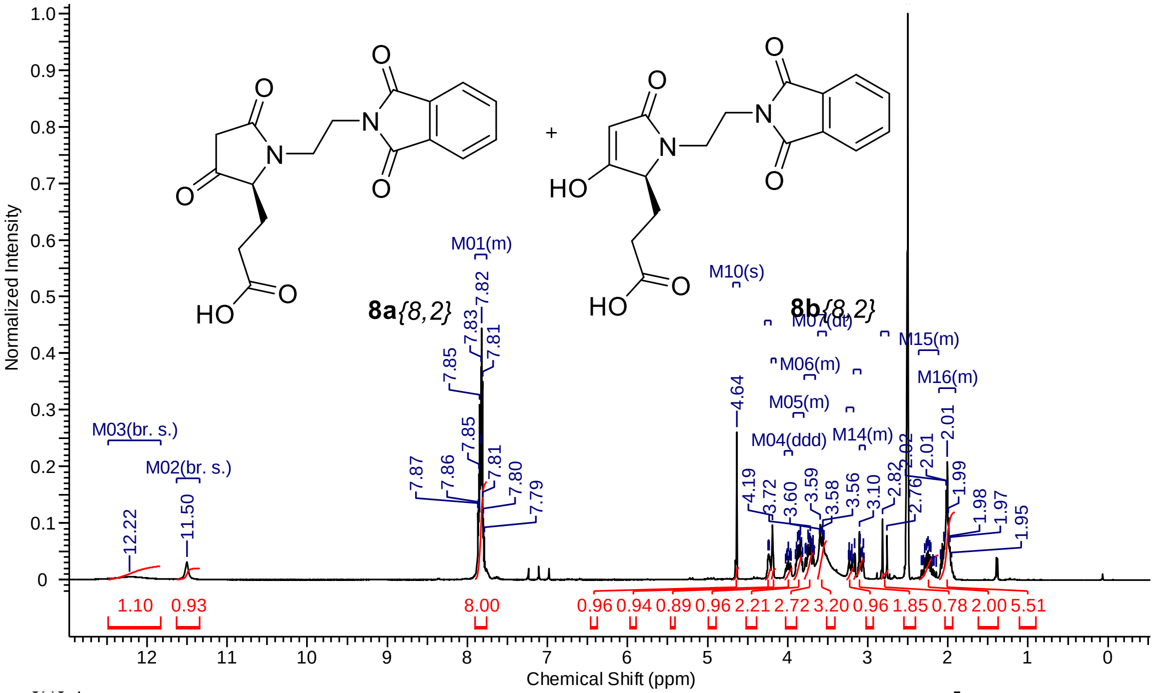

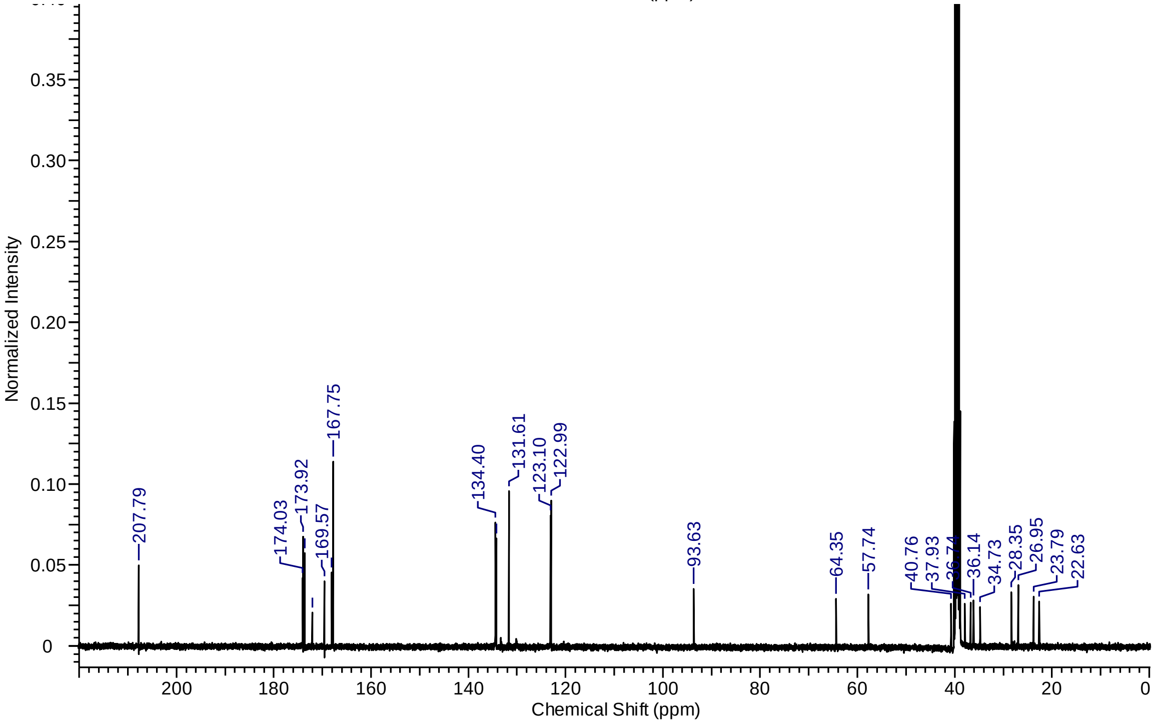

^{1}H and ^{13}C NMR spectra ($CDCl_3$) of (*S*)-3-(1-(2-(1,3-dioxoisoindolin-2-yl)ethyl)-3,5-dioxopyrrolidin-2-yl)propanoic acid 8a*{8,2}*

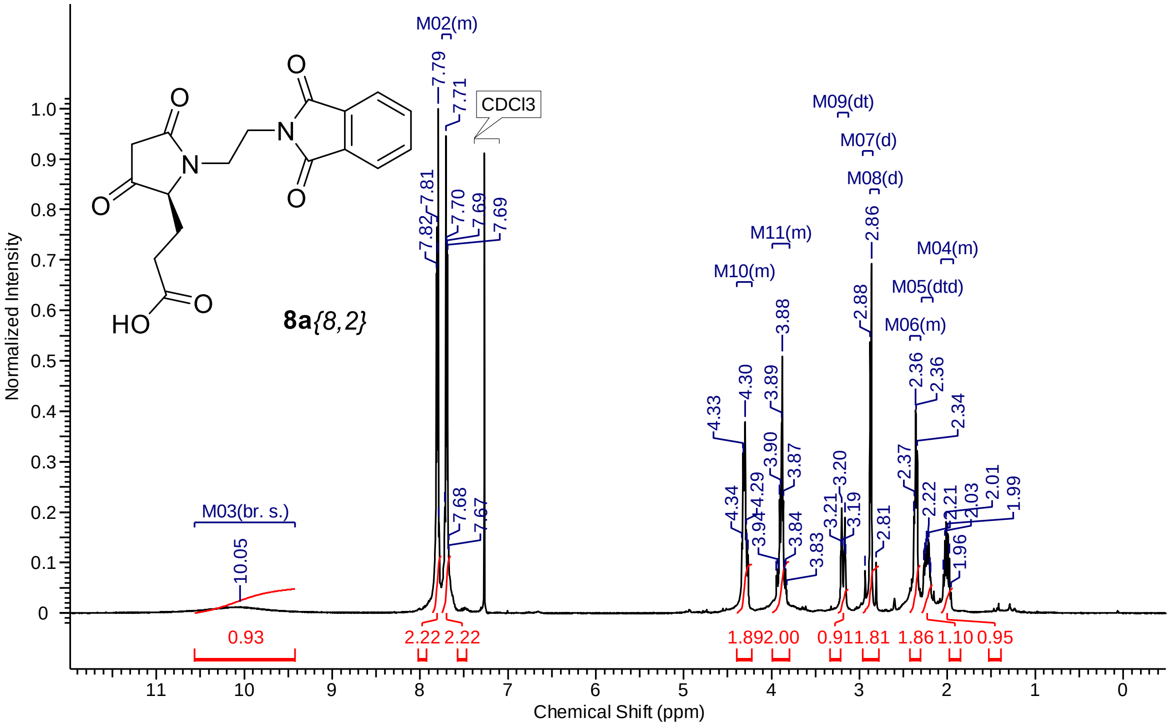

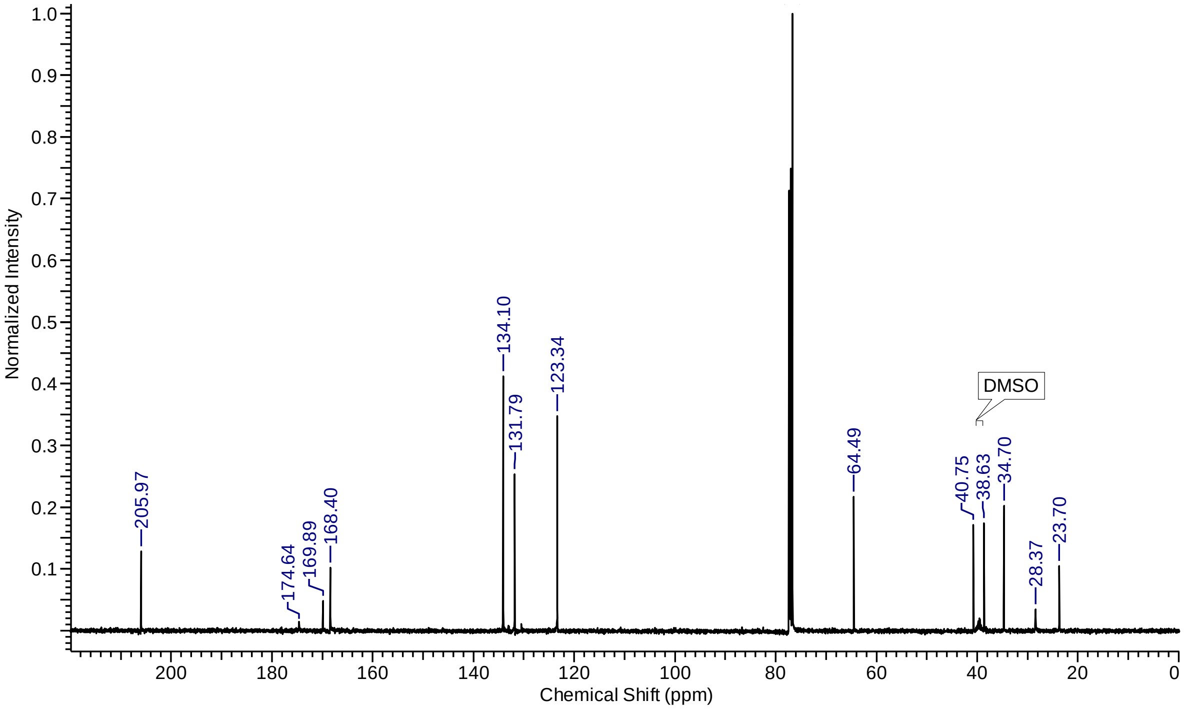

^{1}H and ^{13}C NMR spectra (*d*$_6$-DMSO) of ammonium 1,1'-dimethyl-2,5'-dioxo-2,2',5,5'-tetrahydro-1*H*,1'*H*-[3,3'-bipyrrol]-4-olate 9b*{1,1}*

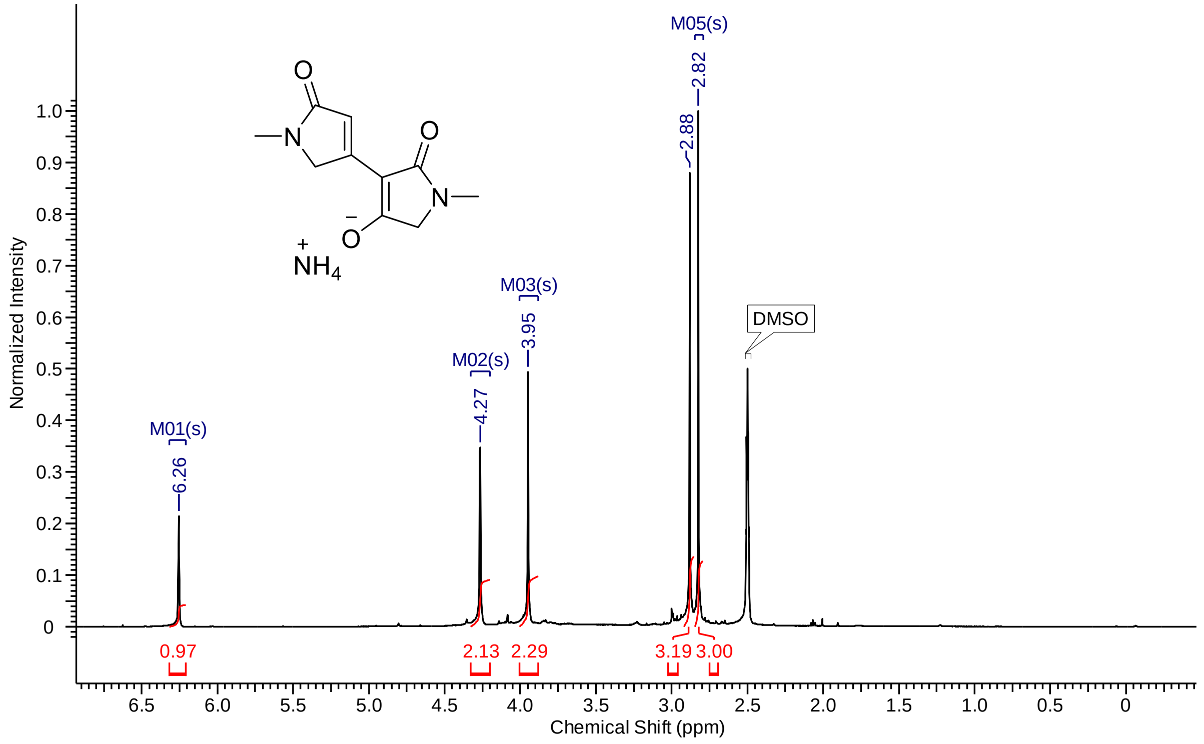

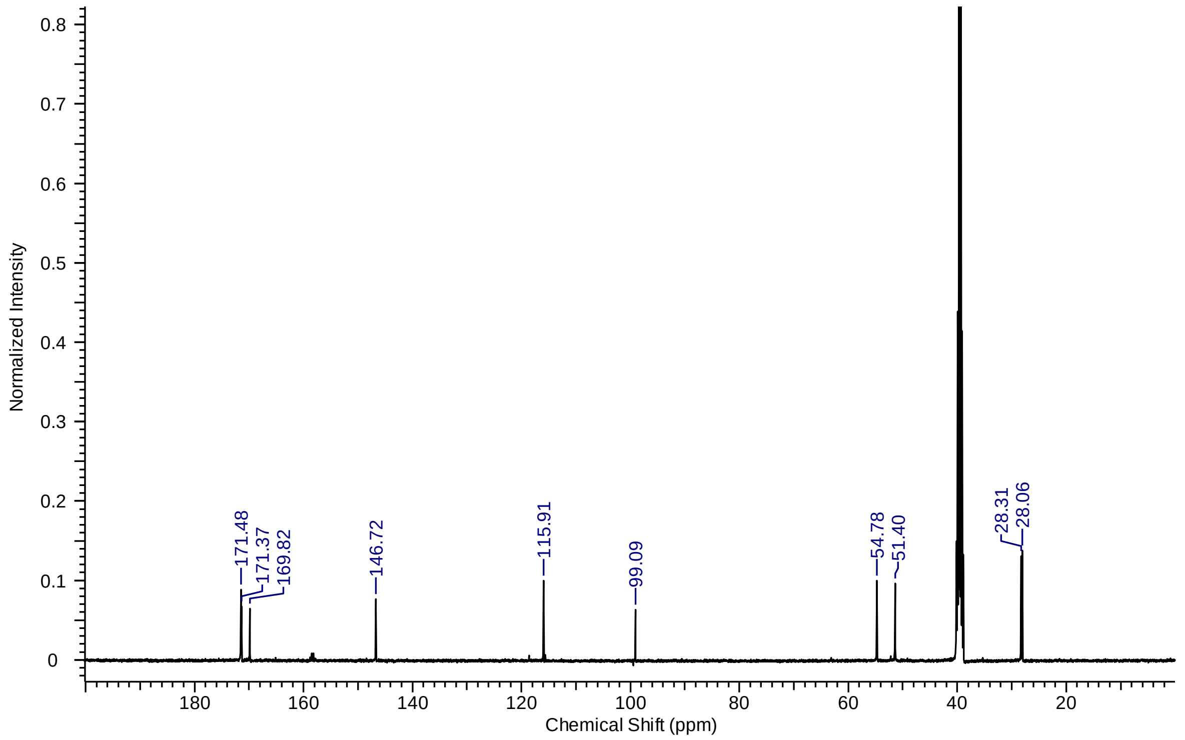

^{1}H and ^{13}C NMR spectra (*d*$_6$-DMSO) of ammonium 1,1'-bis(2-(1,3-dioxoisoindolin-2-yl)ethyl)-2,5'-dioxo-2,2',5,5'-tetrahydro-1*H*,1'*H*-[3,3'-bipyrrol]-4-olate 9b*{1,2}*

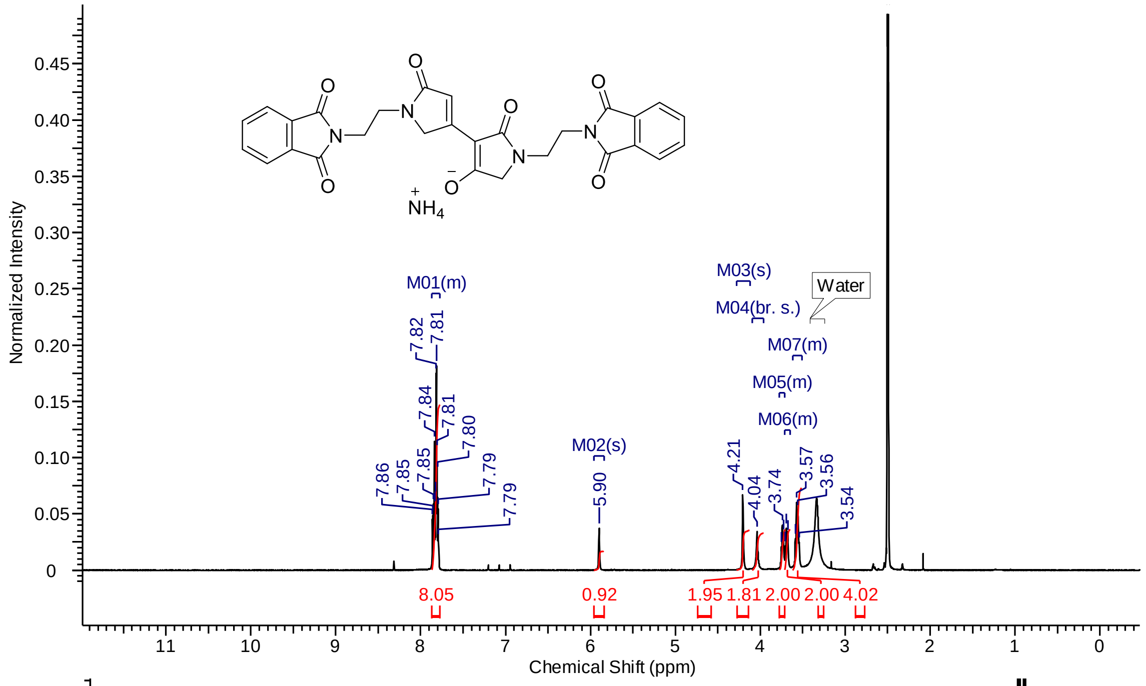

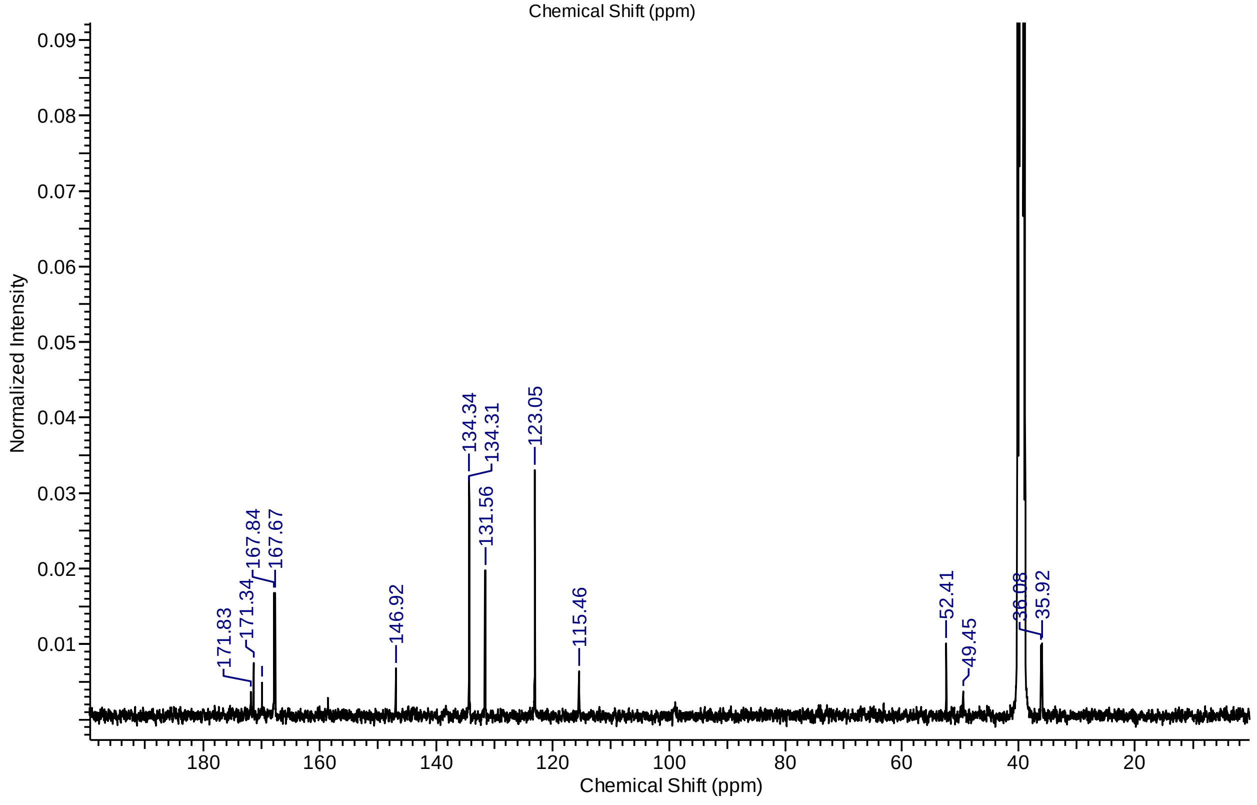

^{1}H and ^{13}C NMR spectra (d_6-DMSO) of ammonium (6*S*,6'*R*,7aS,7'a*S*)-6,6'-bis(benzyloxy)-3,3'-dioxo-5,5',6,6',7,7a,7',7'a-octahydro-3*H*,3'*H*-[1,2'-bipyrrolizin]-1'-olate 9b*{2,-}*

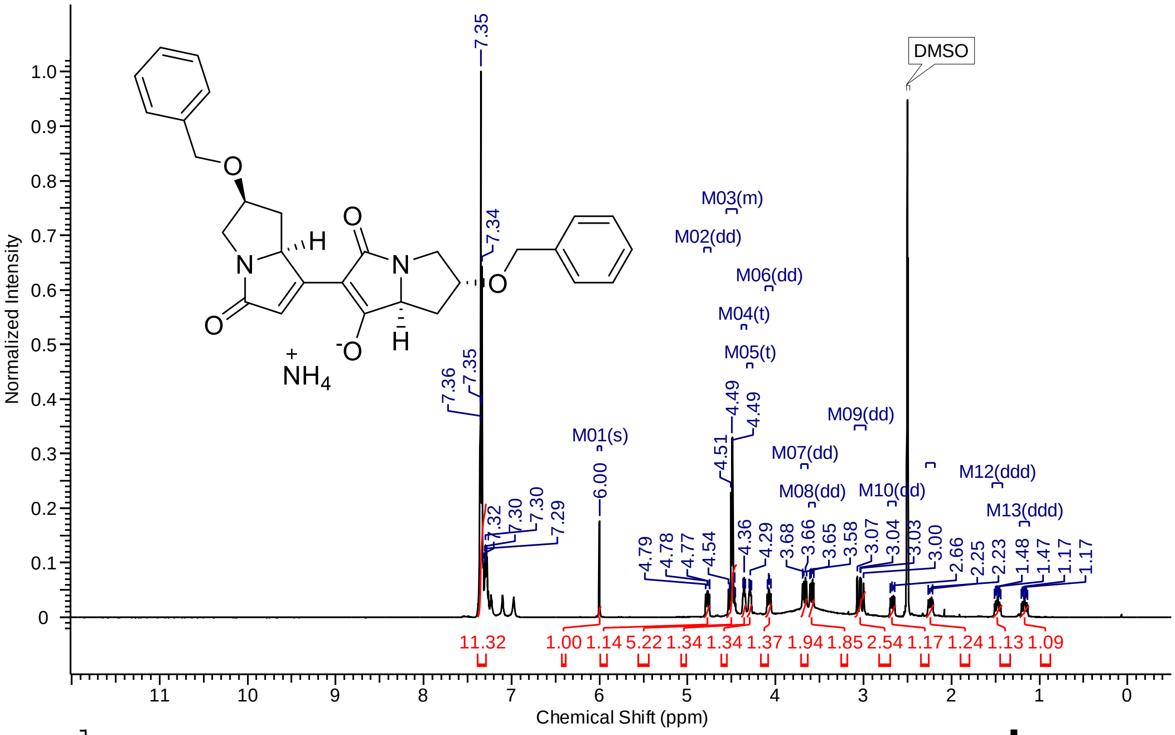

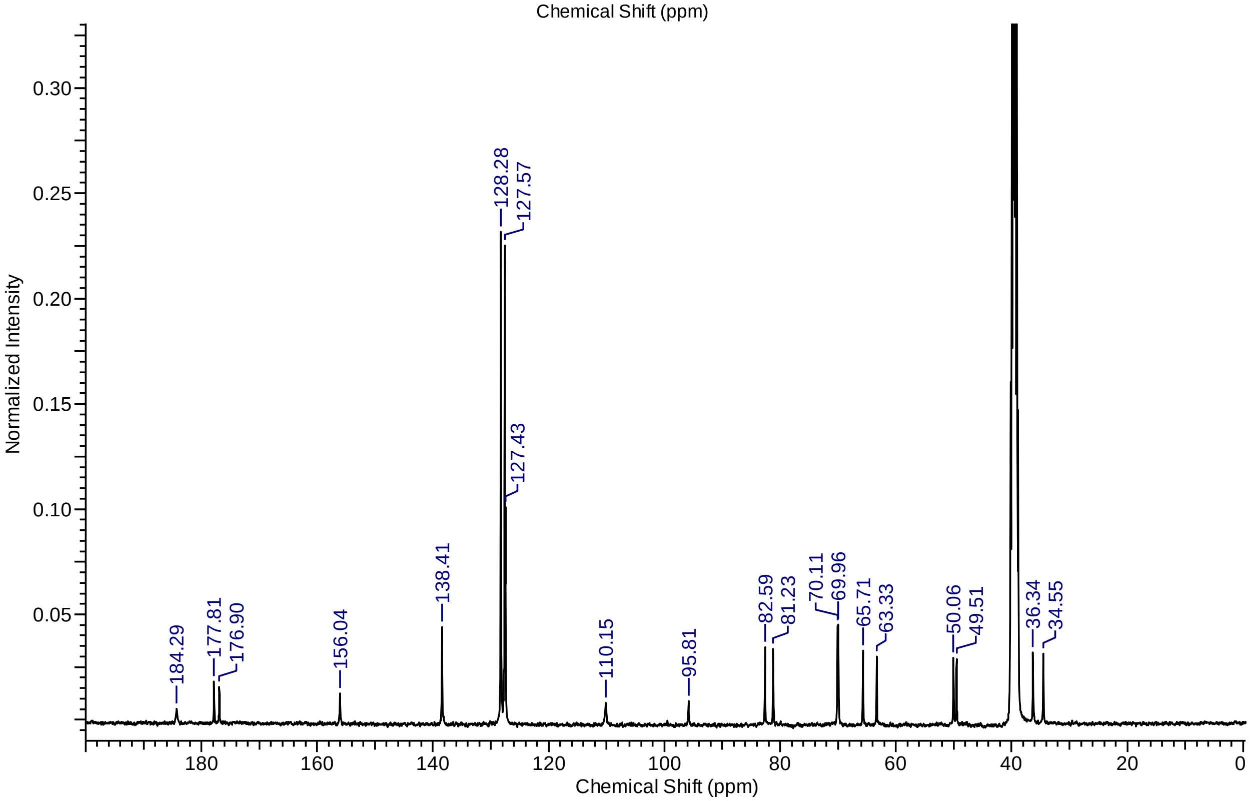

^{1}H and ^{13}C NMR spectra (d_6-DMSO) of ammonium (2'*S*,5*S*)-1,1'-dibenzyl-2',5-dimethyl-2,5'-dioxo-2,2',5,5'-tetrahydro-1*H*,1'*H*-[3,3'-bipyrrol]-4-olate 9b*{5,3}*

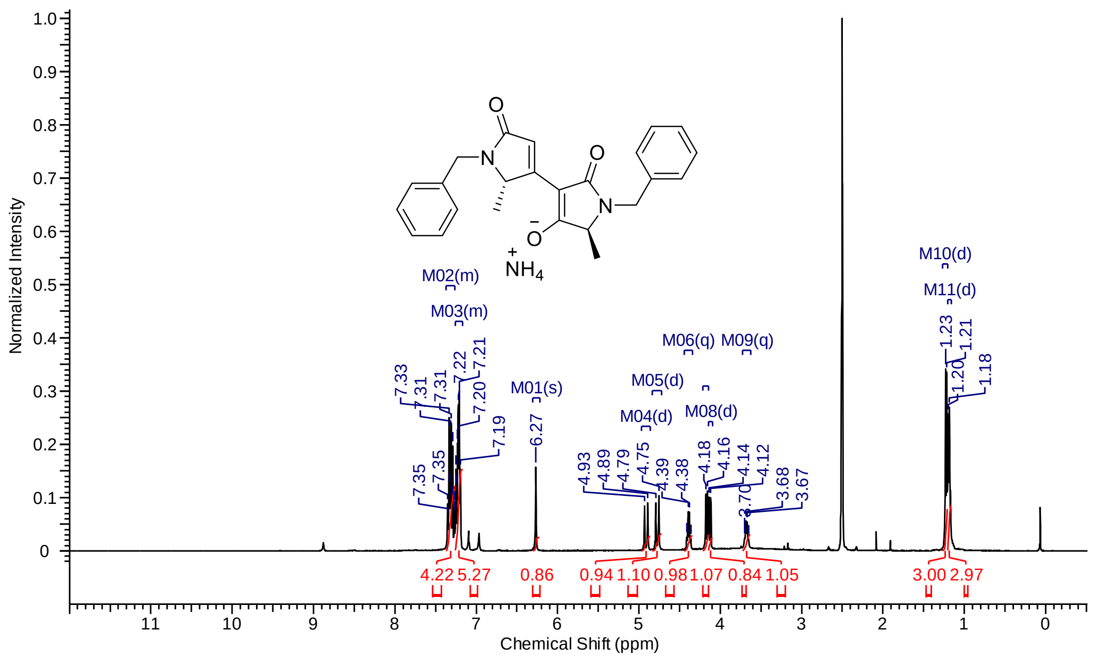

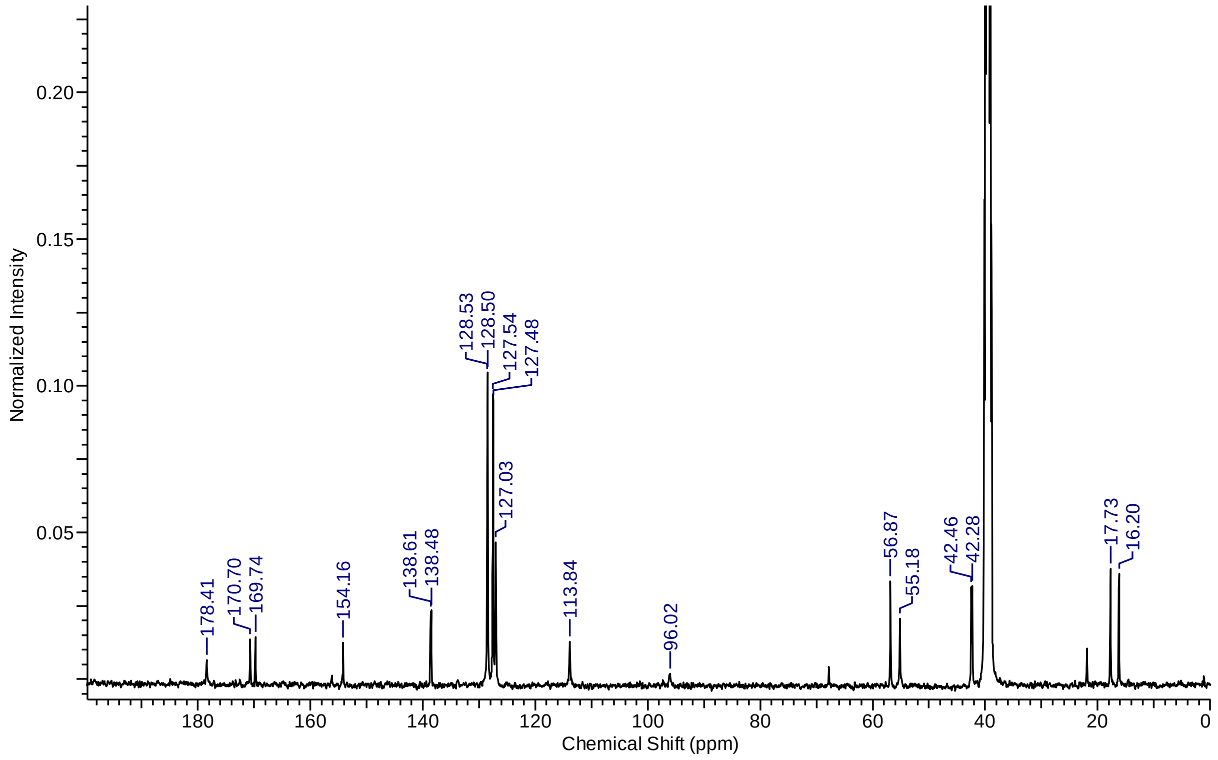

^{1}H and ^{13}C NMR spectra (*d$_6$*-DMSO) of ammonium (2'*S*,5*S*)-2',5-dibenzyl-1,1'-bis(2-(1,3-dioxoisoindolin-2-yl)ethyl)-2,5'-dioxo-2,2',5,5'-tetrahydro-1*H*,1'*H*-[3,3'-bipyrrol]-4-olate 9b*{6,2}*

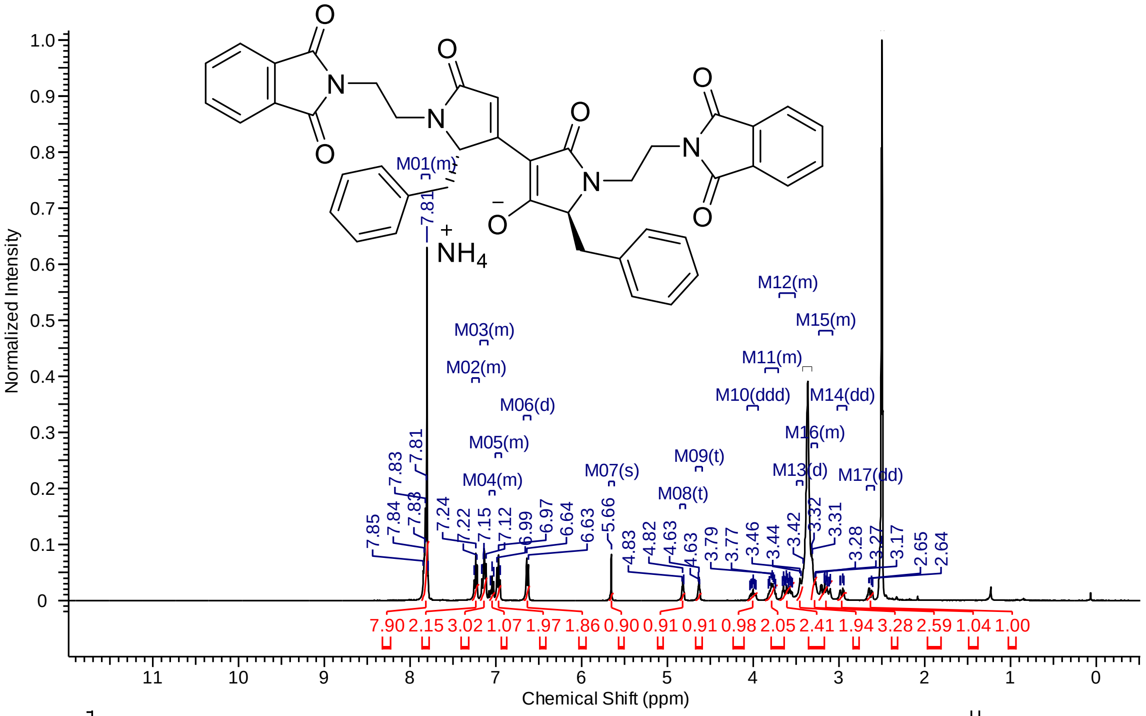

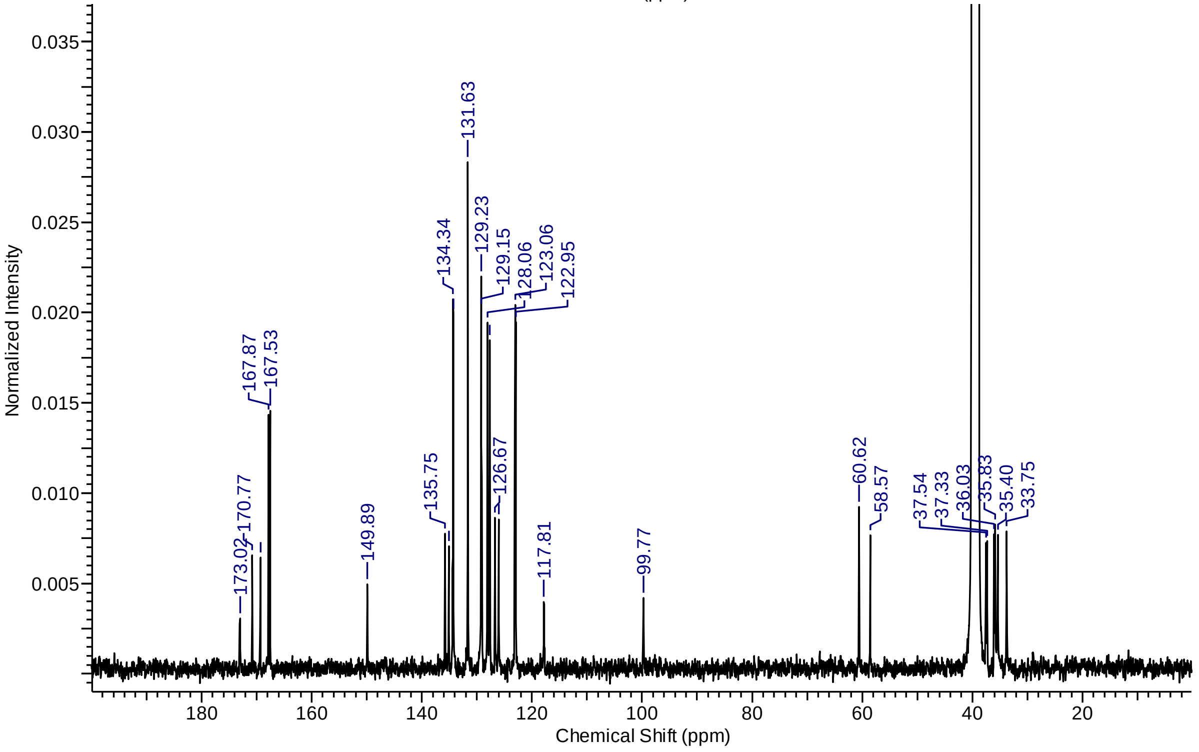

Supplement: Supplementary file 1 [file molecules-24-01406-s001.pdf]
